# Supplementary material for: Generating active metal/oxide reverse interfaces through coordinated migration of single atoms
Source: Nat Commun. 2024 Feb 9;15:1234. doi: 10.1038/s41467-024-45483-w (PMC10858022; doi:10.1038/s41467-024-45483-w)
Supplement: Supplementary file 1 — Supplementary Information [file 41467_2024_45483_MOESM1_ESM.pdf]

Supplementary Information for

Generating Active Metal/Oxide Reverse Interfaces through Coordinated Migration of Single Atoms

Lina Zhang[1,2]†, Shaolong Wan[1]†, Congcong Du[1]†, Qiang Wan[3]†, Hien Pham[4], Jiafei Zhao[1], Xingyu Ding[1], Diye Wei[1], Wei Zhao[5], Jiwei Li[1], Yanping Zheng[1], Hui Xie[1], Hua Zhang[1], Mingshu Chen[1], Kelvin H.L. Zhang[1], Shuai Wang[1,2], Jingdong Lin[1], Jianyu Huang[6], Sen Lin[3]*, Yong Wang[7], Abhaya K. Datye[4], Ye Wang[1,2]*, and Haifeng Xiong[1,2]*

[1] State Key Laboratory of Physical Chemistry of Solid Surfaces, Collaborative Innovation Center of Chemistry for Energy Materials, College of Chemistry & Chemical Engineering, Xiamen University; Xiamen, 361005, China

[2] Innovation Laboratory for Sciences and Technologies of Energy Materials of Fujian Province; Xiamen, 361102, China

[3] State Key Laboratory of Photocatalysis on Energy and Environment, College of Chemistry, Fuzhou University; Fuzhou, 350100, China

[4] Department of Chemical and Biological Engineering and Center for Micro-Engineered Materials, University of New Mexico; Albuquerque, NM 87131, United States

[5] Institute for Advanced Study, Shenzhen University; Shenzhen, 518060, China

[6] Clean Nano Energy Center, State Key Laboratory of Metastable Materials Science and Technology, Yanshan University; Qinhuangdao, 066000, China

[7] Voiland School of Chemical Engineering and Bioengineering, Washington State University; Pullman, WA 99164, United States

† These authors contributed equally to this work.

- Corresponding authors. Email: slin@fzu.edu.cn (S. L.); wangye@xmu.edu.cn (Y. W.); haifengxiong@xmu.edu.cn (H. X.)

This PDF file includes:

Supplementary Figures 1-49

Supplementary Tables 1-10

References 1-10

Supplementary Table 1. Comparison of the performance of Pd/CeO_2-AT-S with other Pd-based catalysts reported in the literature in HCHO oxidation.

**Catalyst**	**Metal loading (wt%)**	**Reaction condition**	T_{100} [a] (°C)	r (μmol·g_m^{-1}·s^{-1})	**Ref.**
Pd/CeO_2-AT-S	1.0	400 ppm HCHO, 20% O_2, N_2 balance	25	100	This work
Pd/TiO_2-600	1.0	150 ppm HCHO, 20% O_2, N_2 balance, RH = 35%	25	53	[1]
Pd/nano-γ-Al_2O_3	1.0	120 ppm HCHO, 20% O_2, N_2 balance	25	72	[2]
2Na-1Pd/TiO_2	1.0	300 ppm HCHO, 20% O_2, N_2 balance, RH = 30%	RT	30.0	[3]
Pd/CeO_2-Cube	0.96	600 ppm HCHO, 20% O_2, N_2 balance	RT	21.6	[4]
Pd/CeO_2	0.78	600 ppm HCHO, 20% O_2, N_2 balance	RT	-	[5]

[a] T_{100}: The temperatures at 100% conversion of HCHO

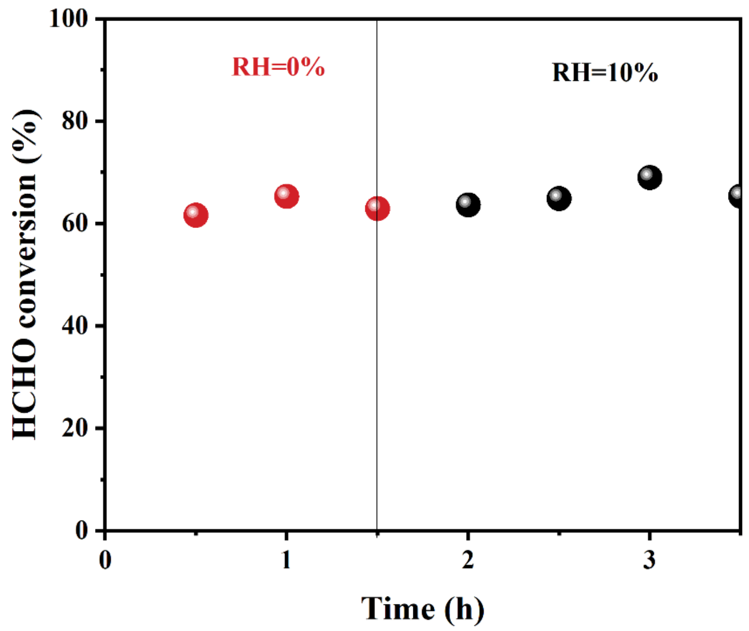

Supplementary Fig. 1. Relative humidity effect on the activity of Pd/CeO_2-AT-S catalyst at 30℃. Reaction conditions: 400 ppm HCHO, 20 vol% O_2, and N_2 as balance gas, total flow rate: 50 mL·min^{-1} and WHSV: 176,000 mL·g^{-1}·h^{-1}.

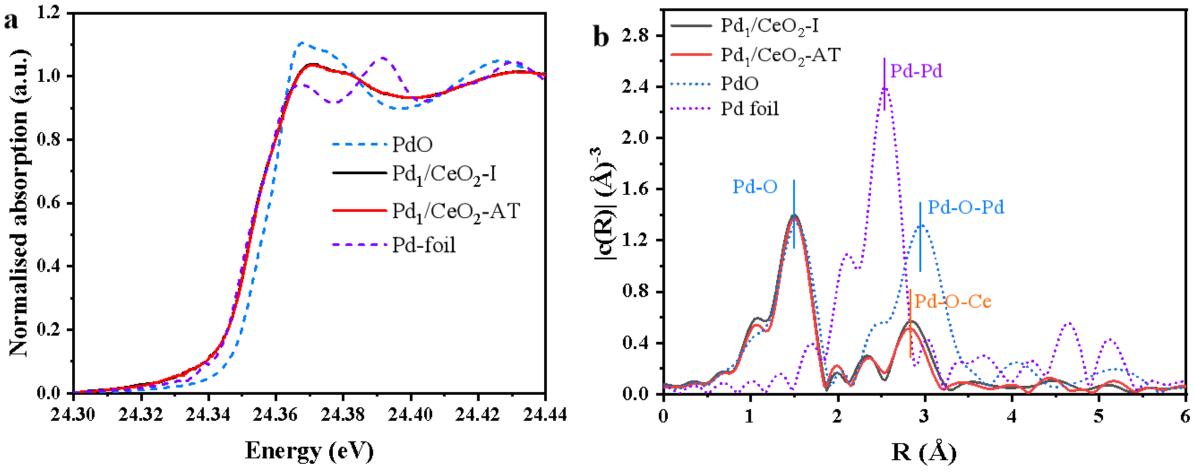

Supplementary Fig. 2. Pd K-edge XANES (**a**) and the k^3-weighted FT-EXAFS spectra in the R-space (**b**) of the Pd-foil, PdO, Pd_1/CeO_2-AT and Pd_1/CeO_2-I catalysts (Fitting details are reported in **Supplementary Table 2**).

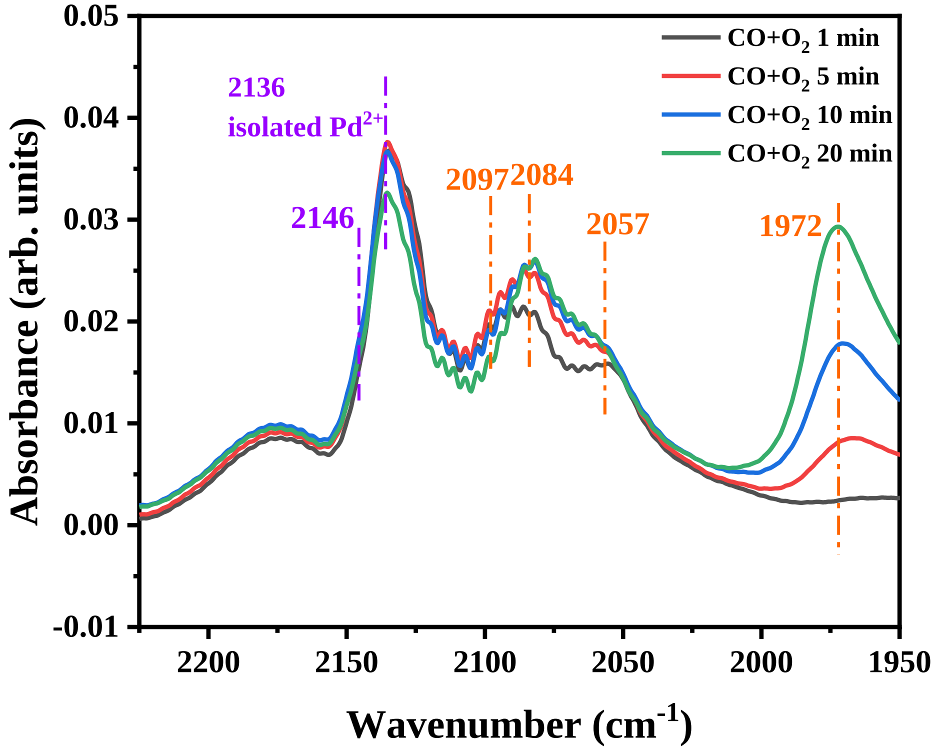

Supplementary Fig. 3. CO-DRIFTS spectra of the Pd/CeO_2-AT catalyst after exposure to a flow $CO/O_2/N_2$ for 20 min at 125℃.

Supplementary Note: As shown in Supplementary Fig. 3, for the Pd_1/CeO_2-AT catalyst, there are initial CO adsorption peaks of 2146 and 2136 cm^{-1} on the isolated Pd^{2+} (i.e., SAs). However, the peak value of CO-Pd_2/Pd_3 adsorption (<2000 cm^{-1}) appeared only after 5 min, which was because some single atom Pd was still reduced after 20 min of CO adsorption. This phenomenon is consistent with the previous report[6]. In addition, based on XAS analysis (Supplementary Fig. 2), we can further confirm that the Pd on the Pd_1/CeO_2-AT catalyst was a single atom.

Supplementary Table 2. The fitting results of EXAFS at Pd K-edge for Pd_1/CeO_2-I and Pd_1/CeO_2-AT catalysts.

**Samples**	**Scattering pair**	**R(Å)**	**Coordination number**	**Debye-Waller Factor (Å^2)**	**ΔE_0 (eV)**	**S_0^2**	**R-factor**
Pd foil	Pd-Pd	2.732	12.0	0.00612	3.539	0.7	0.020717
Pd_1/CeO_2-I	Pd-O	1.980	2.4	0.00099	2.993	0.7	0.018156
	Pd-Ce	3.400	2.3	0.00705	2.993	0.7	
Pd_1/CeO_2-AT	Pd-O	1.983	2.7	0.00239	2.09	0.7	0.035804
	Pd-Ce	3.389	2.3	0.00860	2.09	0.7	
Pd/CeO_2-I-S	Pd-O	1.912	0.3	0.0177	5.11	0.7	0.007969
	Pd-Pd	2.727	6.2	0.00511	5.11	0.7	
Pd/CeO_2-AT-S	Pd-O	1.950	0.4	0.00317	4.289	0.7	0.007412
	Pd-Pd	2.726	5.9	0.00563	4.289	0.7	
Pd/CeO_2-AT-S-O*	Pd-O	1.967	0.6	0.00034	4.754	0.7	0.012457
	Pd-Pd	2.719	5.4	0.00546	4.754	0.7	

*The treated Pd/CeO_2-AT-S catalyst by oxidizing at 250°C for 30 min in air is denoted as Pd/CeO_2-AT-S-O.

Supplementary Table 3. Characterization of the supports and catalysts after different treatments.

Catalysts	Pd loading[a] (wt%)	CeO_2 particle size[b] (nm)	Pd particle size[c] (nm)	Surface area ($m^2 \cdot g^{-1}$)	Pore volume[d] ($cm^3 \cdot g^{-1}$)	Average Pore size[e] (nm)
CeO_2	0	9	NA[f]	80	0.22	11
CeO_2-S[g]	0	28	NA	12	0.05	16
Pd_1/CeO_2-AT	1.06	25	Atomic dispersed	26	0.10	15
Pd/CeO_2-AT-Ar	1.0	25	ND[f]	25	0.10	11
Pd/CeO_2-AT-S	1.05	26	10	25	0.10	14
Pd_1/CeO_2-I	1.0	9	Atomic dispersed	59	0.13	10
Pd/CeO_2-I-S	1.0	23	32	14	0.07	17

[a] Determined by the ICP-OES technique.

[b] Determined by the XRD results according to the Scherrer equation using FWHM of the (111) peak of CeO_2.

[c] Determined by the XRD results according to the Scherrer equation using FWHM of the (111) peak of Pd.

[d] Total pore volume at a P/P_o ratio of 0.99.

[e] Average pore size obtained from desorption curve.

[f] ND = not detected, NA = not available.

[g] Steam treated in 10 v/v% H_2O/Ar at 750℃.

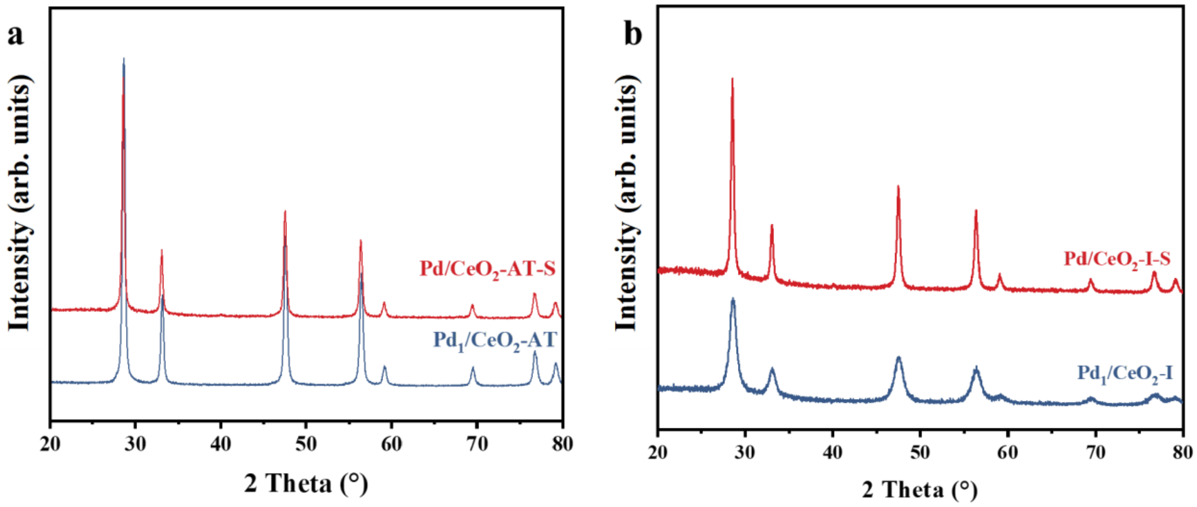

Supplementary Fig. 4. XRD patterns for the (**a**) Pd/CeO_2-AT-S and Pd_1/CeO_2-AT catalysts and (**b**) Pd/CeO_2-I-S and Pd_1/CeO_2-I catalysts performed at a scanning speed of 2θ = 10 °/min from 20° to 80°. These wide scanning spectra only show the diffraction peaks of crystallite CeO_2.

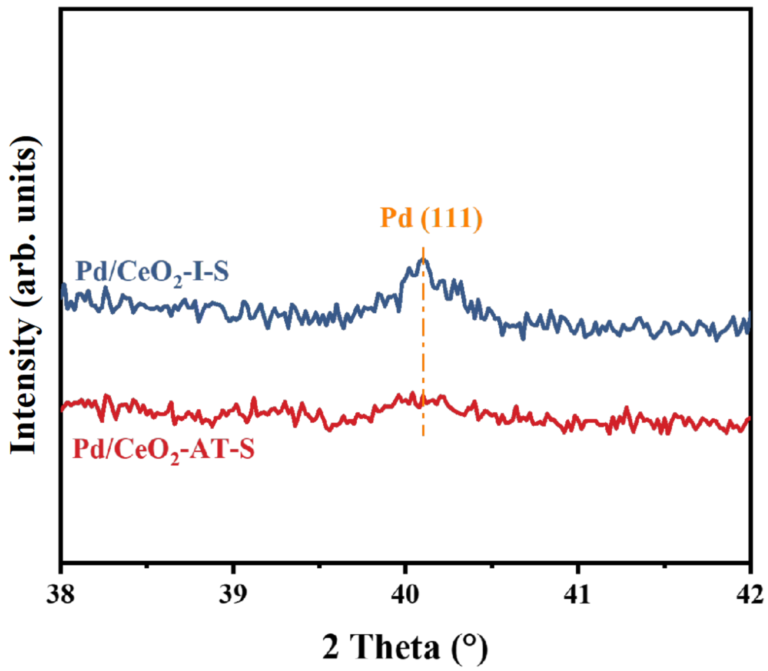

Supplementary Fig. 5. XRD patterns for the Pd/CeO_2-I-S and Pd/CeO_2-AT-S catalysts performed at a scanning speed of 2θ = 0.6 °/min from 38° to 42°. The presence of diffraction peaks on the narrow scanning demonstrates the formation of Pd nanoparticles for the Pd single-atom catalysts after steam treatment.

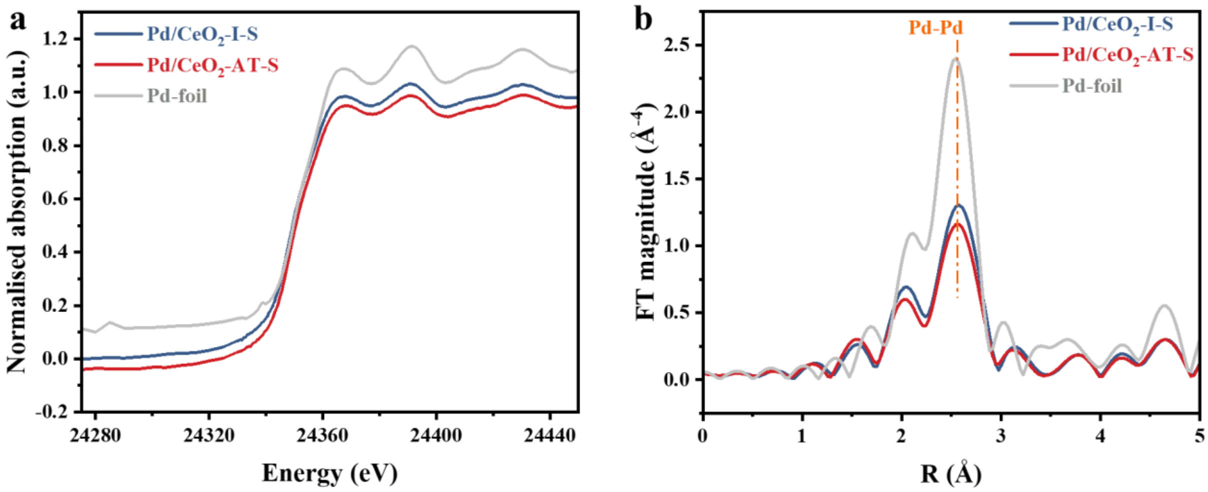

Supplementary Fig. 6. Pd K-edge XANES (**a**) and the k^3-weighted FT-EXAFS spectra in the R-space (**b**) of the Pd-foil, Pd/CeO_2-AT-S and Pd/CeO_2-I-S catalysts (Fitting details are reported in **Supplementary Table 2**. It confirms the formation of Pd nanoparticles after treating Pd single-atom catalysts in steam (750°C).

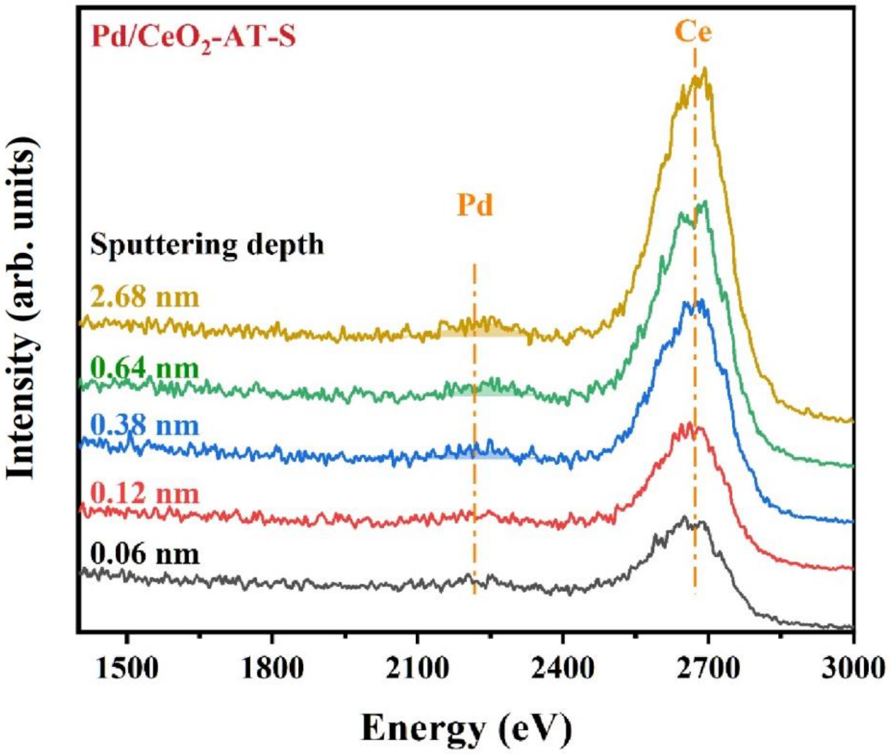

Supplementary Fig. 7. 5 keV $^{20}Ne^{+}$ HS-LEIS spectra of the Pd/CeO_2-AT-S catalyst showing the surface species at different sputtering depths. More Pd signal was detected with the increase of the sputtering depth indicating the coverage of Pd nanoparticle by other surface species.

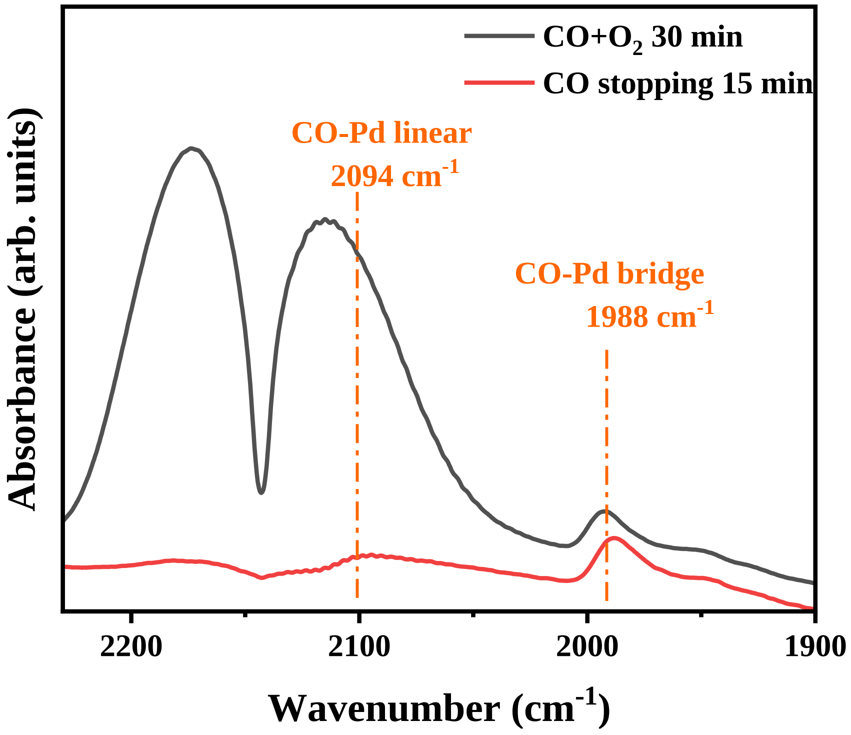

Supplementary Fig. 8. CO-DRIFTS spectra of the Pd/CeO_2-I-S catalyst after exposure to a flow of CO/O_2/N_2 for 30 min and degassing in O_2/N_2 for 15 min at 30℃. It indicates the formation of metal Pd nanoparticle on the steam-treated catalyst.

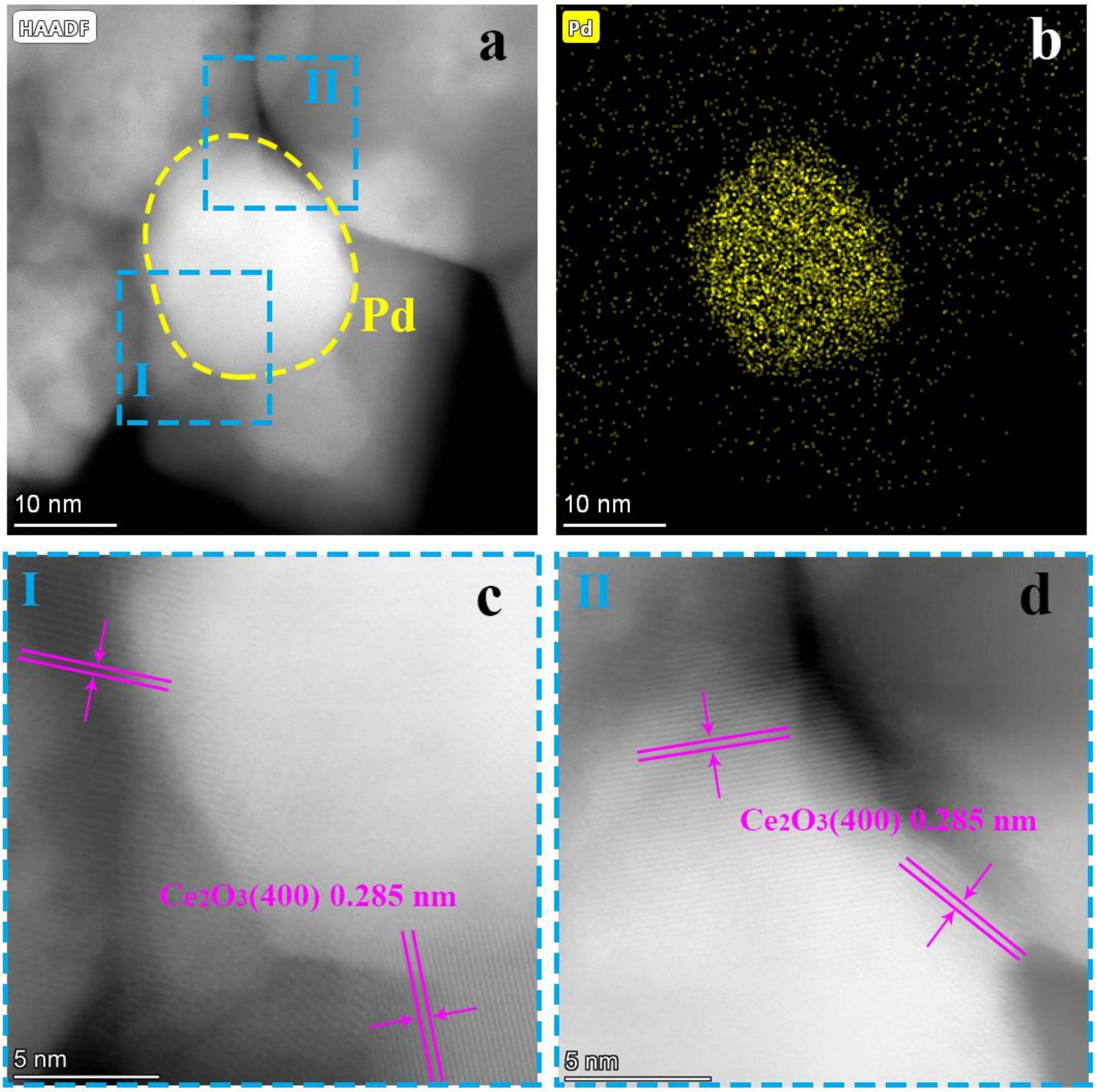

Supplementary Fig. 9. HAADF-STEM (**a, c, d**) images and STEM-EDS mapping (**b**) of the Pd/CeO_2-AT-S catalyst. (**c, d**) High-resolution lattice fringes of the rectangular region in **Supplementary Fig. 9a**.

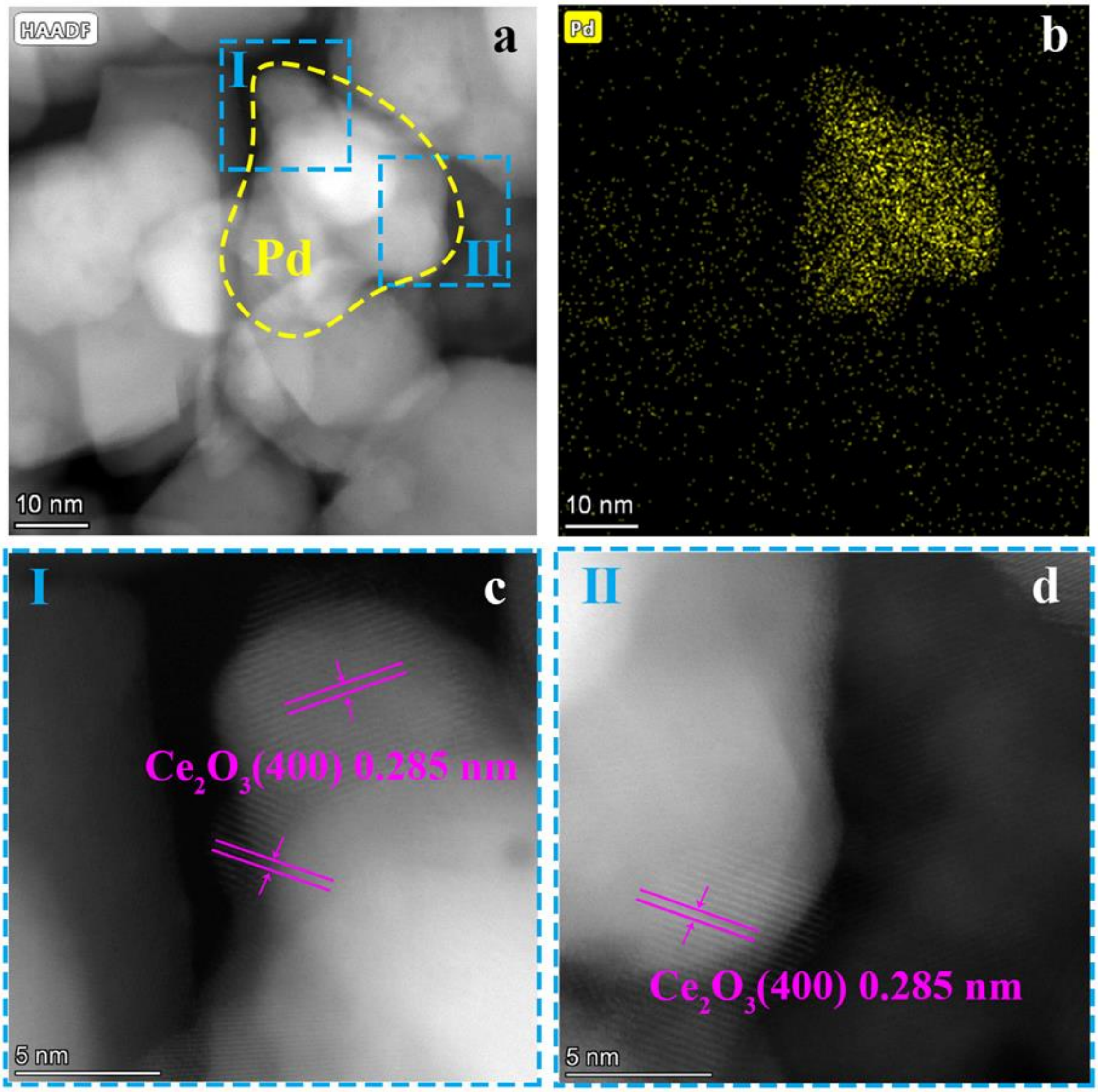

Supplementary Fig. 10. HAADF-STEM (**a, c, d**) images and STEM-EDS mapping (**b**) of the Pd/CeO_2-AT-S catalyst. (**c, d**) High-resolution lattice fringes of the rectangular region in **Supplementary Fig. 10a**.

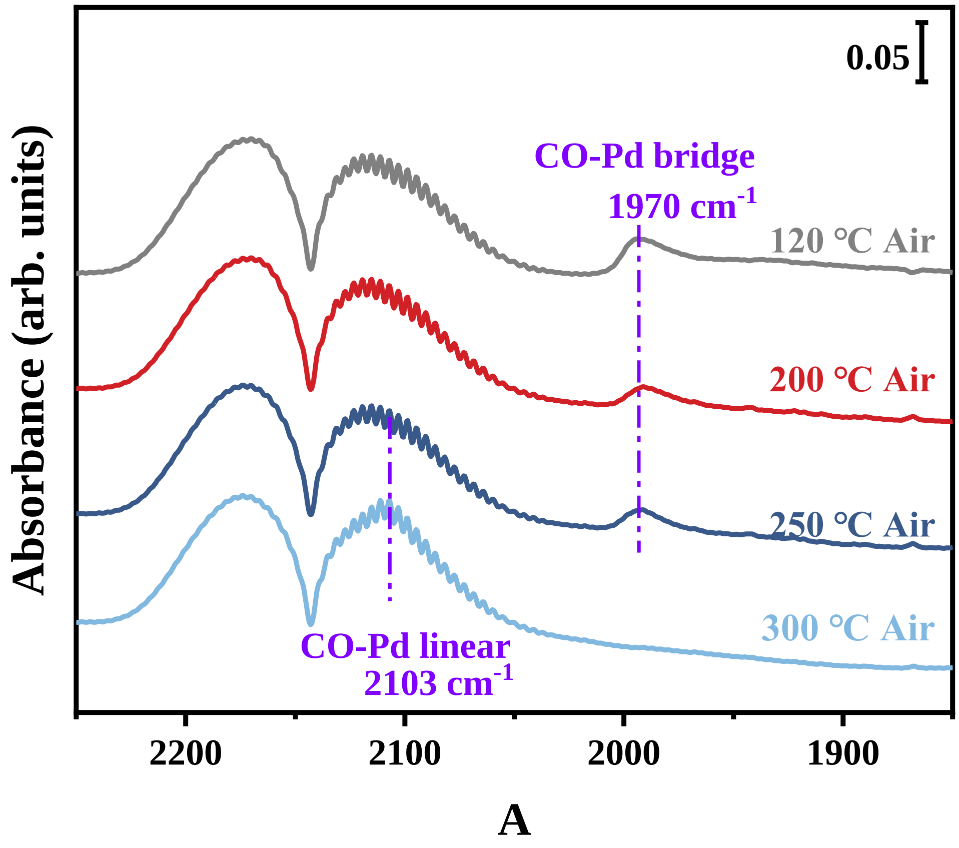

Supplementary Fig. 11. CO-DRIFTS spectra of the Pd/CeO_2-AT-S catalyst treated at different temperatures in air after exposure to a flow of CO/O_2/N_2 for 30 min at 30°C.

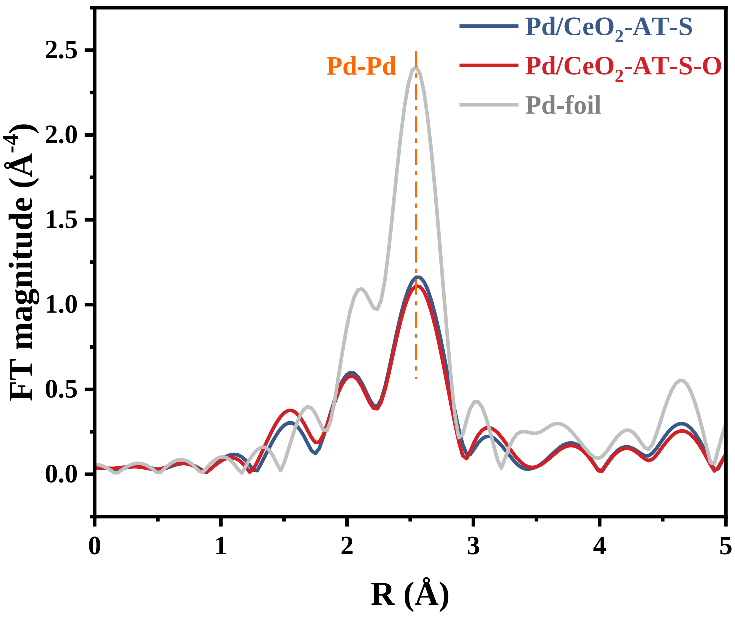

Supplementary Fig. 12. The k^3-weighted FT-EXAFS spectra (Pd K edge) of the Pd-foil, Pd/CeO_2-AT-S and Pd/CeO_2-AT-S-O catalysts (The catalyst of Pd/CeO_2-AT-S after oxidizing at 250℃ for 30 min in air is denoted as Pd/CeO_2-AT-S-O, the fitting details are reported in **Supplementary Table 2**). The result shows that the Pd nanoparticle on Pd/CeO_2-AT-S maintains as metal after oxidation at 250℃.

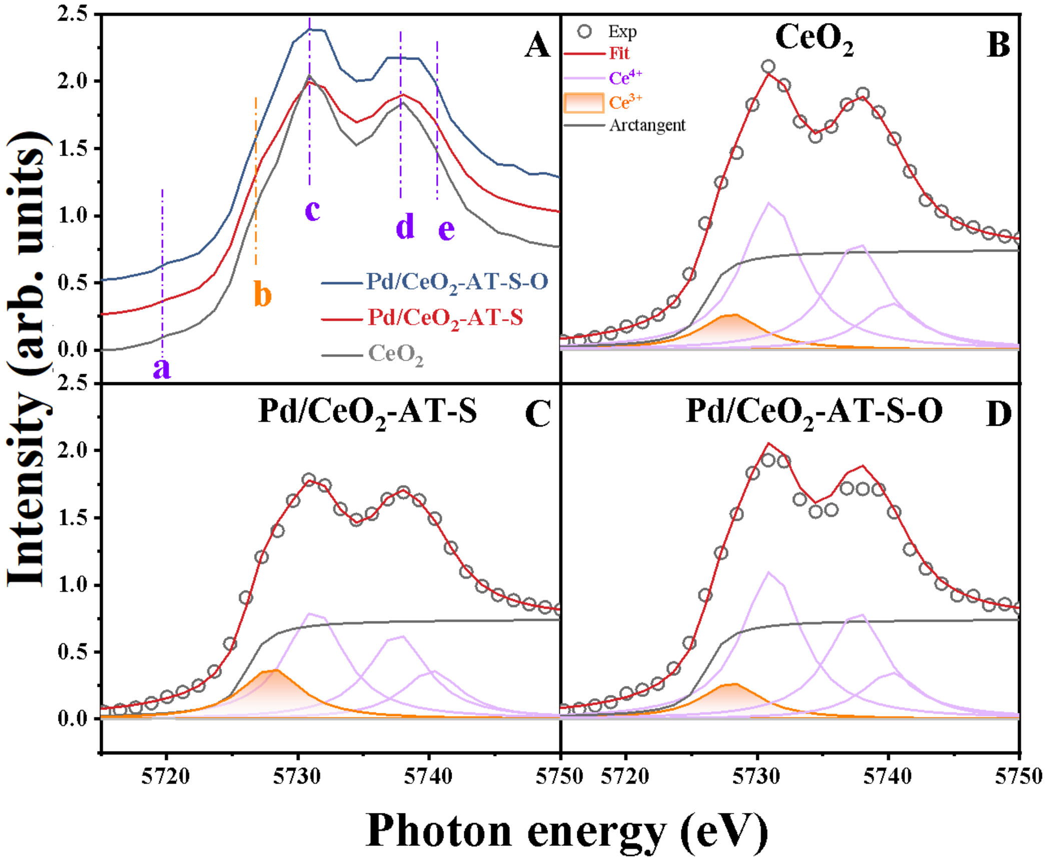

Supplementary Fig. 13. (**a**) Ce L_3-edge spectra of CeO_2, Pd/CeO_2-AT-S, and Pd/CeO_2-AT-S-O catalysts, and (**b, c, d**) is the Gaussian fit of Ce L_3 XANES spectra. Circles and solid lines represent the data and the fit, respectively. (The treated Pd/CeO_2-AT-S catalyst by oxidizing at 250°C for 30 min in air is denoted as Pd/CeO_2-AT-S-O).

Supplementary Table 4. The fitting results of the XANES of the Ce L_3-edge. The photon energy (eV) and the area of each peak is reported.

Peaks		Samples		
Photon energy (eV)		Pd/CeO_2-AT-S	Pd/CeO_2-AT-S-O	CeO_2
**A**	**5717.8**	0.022	0.052	0.014
**B**	**5727.3**	3.251	3.375	2.315
**C**	**5730.8**	6.990	8.413	9.543
**D**	**5738.1**	5.461	5.501	6.895
**E**	**5740.5**	3.103	3.293	2.999
**R-facter**		0.0005	0.0006	0.0006
**Ce^{3+} / (Ce^{4+} + Ce^{3+})**		17.3	16.4	10.6

Peak *B* belongs to Ce^{3+}.

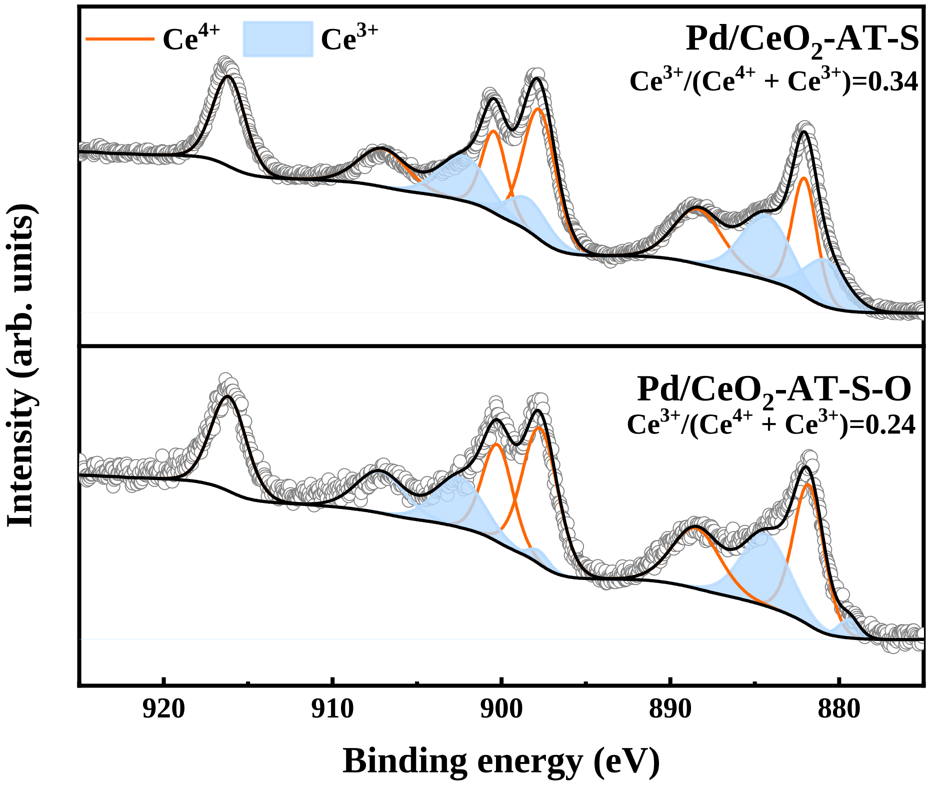

Supplementary Fig. 14. *XPS* spectra of Ce 3*d* for the Pd/CeO_2-AT-S-O and Pd/CeO_2-AT-S catalysts.

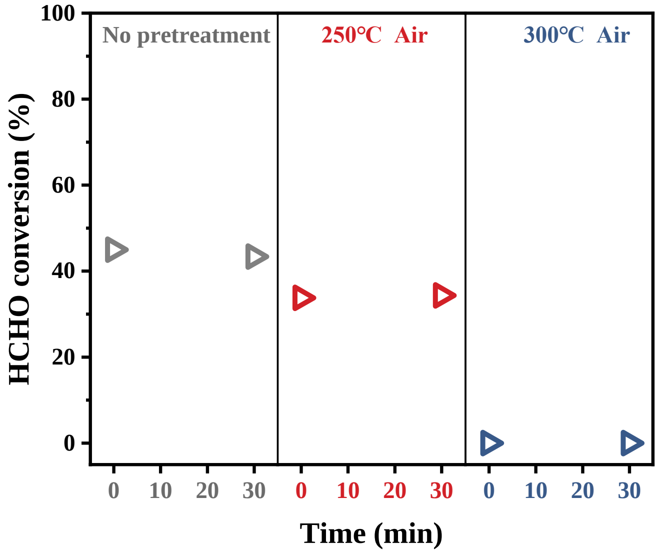

Supplementary Fig. 15. HCHO conversion on the reduced Pd/Al_2O_3 catalyst after treating at different temperatures in air. Reaction condition: 30°C, 400 ppm HCHO, 20 vol% O_2, and N_2 as balance gas, and total flow rate: 50 mL·min^{-1}. The reduced Pd/Al_2O_3 catalyst is active in aldehyde oxidation after treating in air at temperature of ≤ 250°C, while it completely loses activity after treating at 300°C due to the reoxidation of the metal Pd.

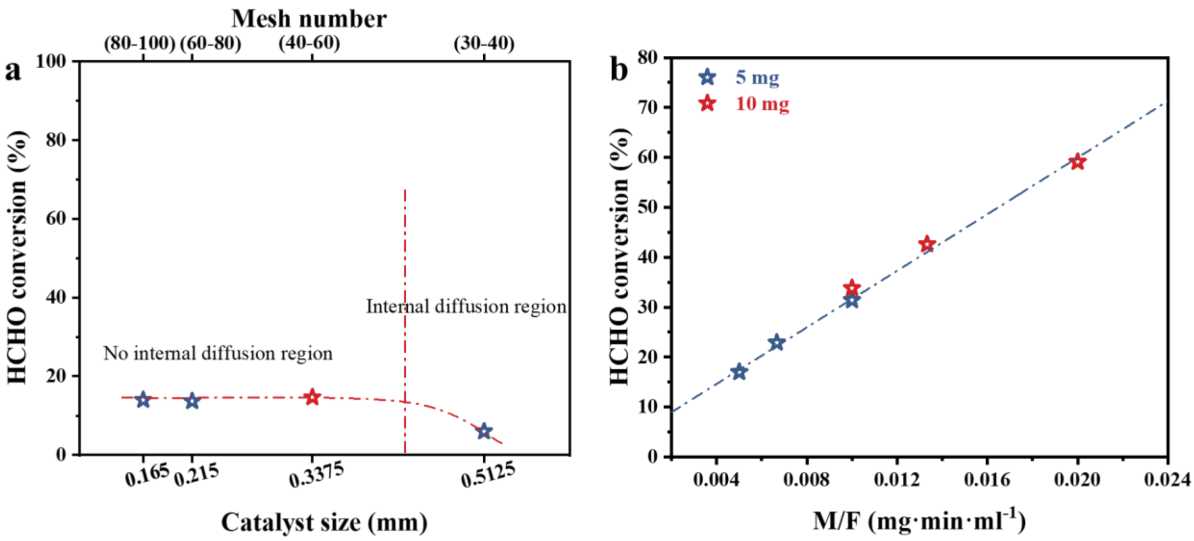

Supplementary Fig. 16. Effect of internal mass transfer (**a**) and external mass transfer (**b**) on the conversion of HCHO to CO_2.

Supplementary Note: We used different particle size catalysts to evaluate the influence of internal mass transfer: 0.425~0.59 mm (mesh 30~40), 0.25~0.425 mm (mesh 40~60), 0.18~0.25 mm (mesh 60~80), and 0.15~0.18 mm (mesh 80~100). As shown in **Supplementary Fig. 16a,** the HCHO conversion was independent of the increased mesh number, which indicated that the internal mass transfer effect can be neglected by using catalyst with a particle size at 0.15~0.425 mm. The catalyst diameter used in this work is in this range. Reaction condition: 400 ppm HCHO, 20 vol% O_2, and N_2 as balance gas, total follow rate = 50 mL·min^{-1}, WHSV: 300,000 mL·g^{-1}·h^{-1}. reaction temperature = 30°C.

We evaluated the effect of external mass transfer by testing HCHO conversion with different catalyst weights (catalyst weight =5 mg and 10 mg, were diluted with ~190 mg of SiO_2, catalyst particle size = 0.25~0.425 mm, flow rate = 50 mL·min^{-1}, 75 mL·min^{-1} and 100 mL·min^{-1}). As shown in **Supplementary Fig. 16b**, a linear relationship between the conversion of HCOH to CO_2 and the ratio of W/F can be observed, suggesting the absence of

external mass transfer resistance. Reaction condition: 400 ppm HCHO, 20 vol% O_2, and N_2 as balance gas, reaction temperature = 30℃.

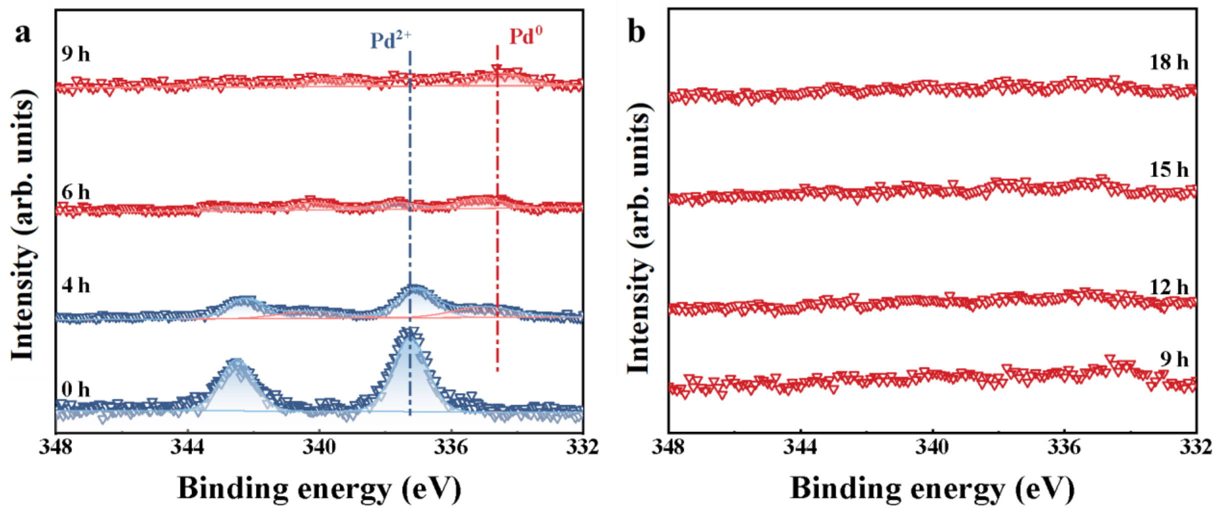

Supplementary Fig. 17. Pd 3*d* *XPS* spectra of the obtained Pd/CeO_2-AT-S catalysts after treating Pd_1/CeO_2-AT catalyst in high-temperature steam for different times (3-18 h). **a,** Pd 3*d* *XPS* spectra after treatment in steam from 0 to 9 h showing the stepwise disappearance of the Pd 3*d* *XPS* spectra. **b,** Pd 3*d* *XPS* spectra after treatment in steam from 9 to 18 h showing the negligible change of the Pd 3*d* *XPS* spectra.

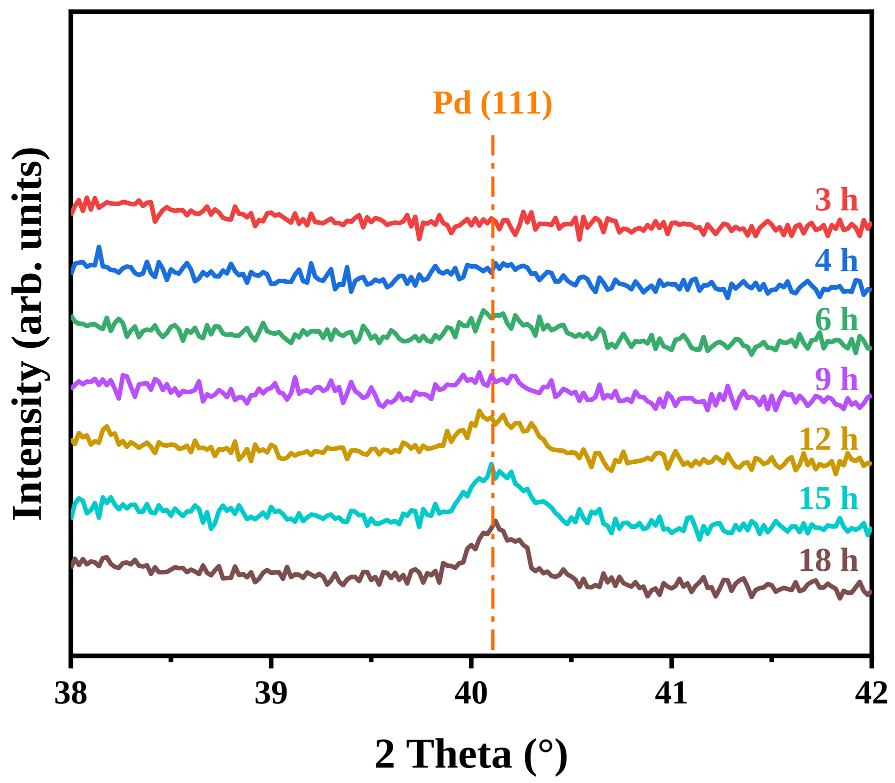

Supplementary Fig. 18. XRD patterns of the obtained Pd/CeO_2-AT-S catalysts after treating Pd_1/CeO_2-AT catalyst in high-temperature steam for different times (3-18 h). XRD was performed at a scanning speed of 2θ = 0.6 °/min from 38° to 42°. The diffraction peak corresponding to the metal Pd (111) becomes sharper with increasing the treatment time in steam, indicating the size of the metal Pd nanoparticle becomes larger.

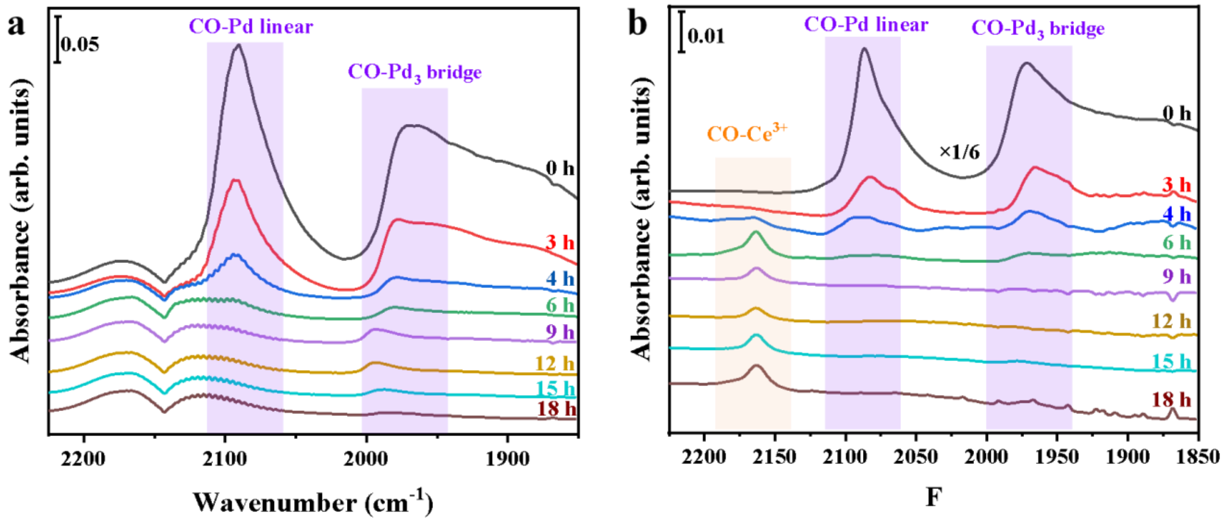

Supplementary Fig. 19. CO-DRIFTS spectra of the Pd/CeO_2-AT-S x h catalyst (**a**) after exposure to a flow CO/O_2/N_2 for 30 min and (**b**) followed by O_2/N_2 degassing for 15 min at 30°C. It indicates that after treatment in steam for 4 h, CO peaks adsorbed on both Ce^{3+} and metal Pd are seen.

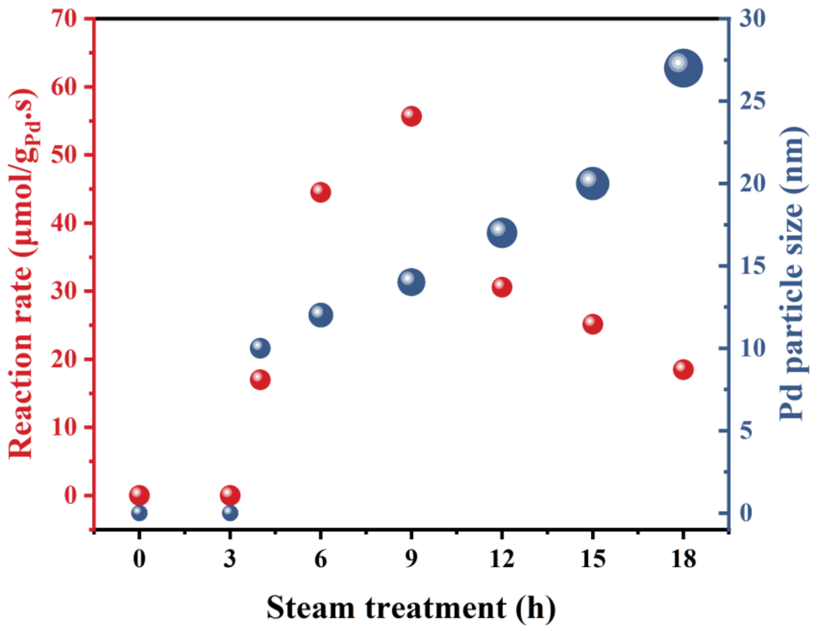

Supplementary Fig. 20. Reaction rate and Pd particle size (calculated from Scherrer equation) of the obtained Pd/CeO_2-AT-S catalysts after treating Pd_1/CeO_2-AT catalyst in high-temperature steam for different times (3-18 h). It indicates the positive relation of the Pd particle size and the treatment time in steam, while the volcano feature is found for the relationship between reaction rate and steam treatment time.

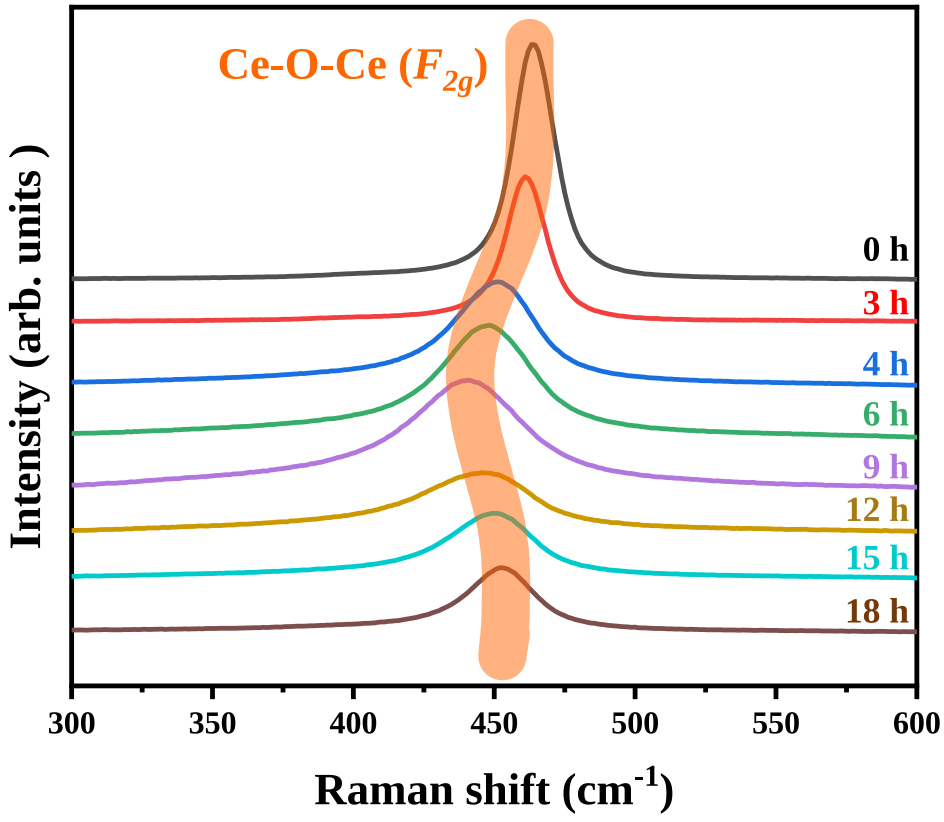

Supplementary Fig. 21. Raman spectra of the obtained Pd/CeO_2-AT-S catalyst after treating Pd_1/CeO_2-AT SAC in high-temperature steam for different times (3-18 h) showing the shift of Ce-O-Ce band with treatment time.

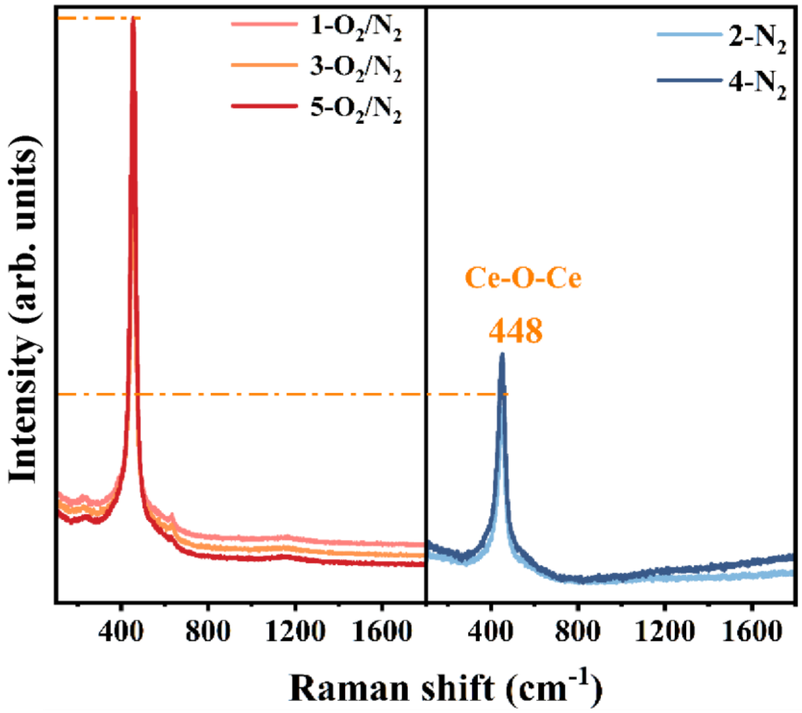

Supplementary Fig. 22. *In situ* Raman spectra of the Pd/CeO_2-AT-S catalyst cycled in different atmospheres (O_2/N_2 or N_2) at 120°C.

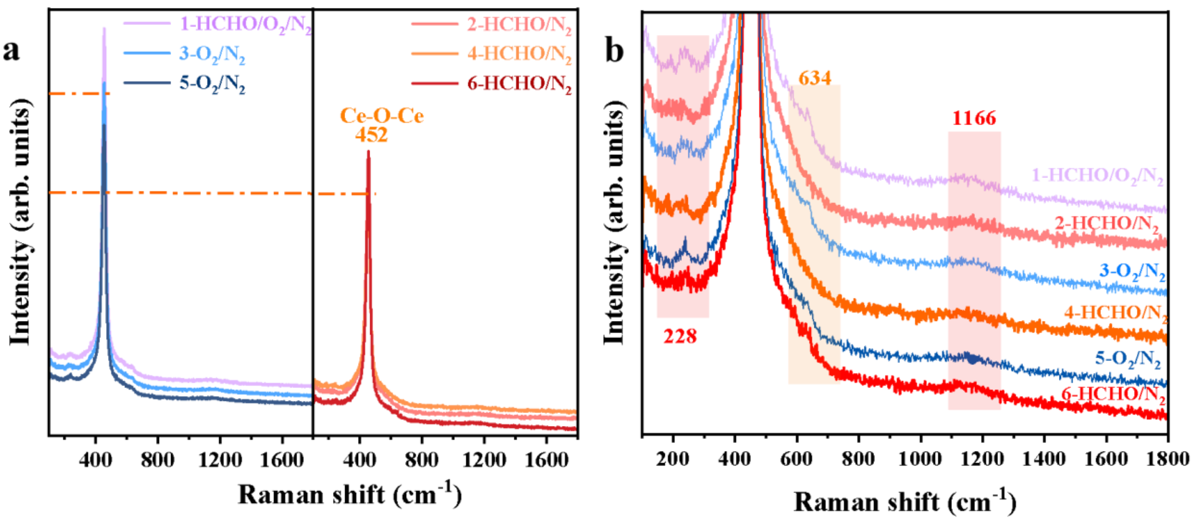

Supplementary Fig. 23. *In situ* Raman spectra (**a**) and the magnified spectra (**b**) of the Pd/CeO_2-AT-S catalyst cycled in different atmospheres (HCHO/O_2/N_2, HCHO/N_2 or O_2/N_2) at 25°C.

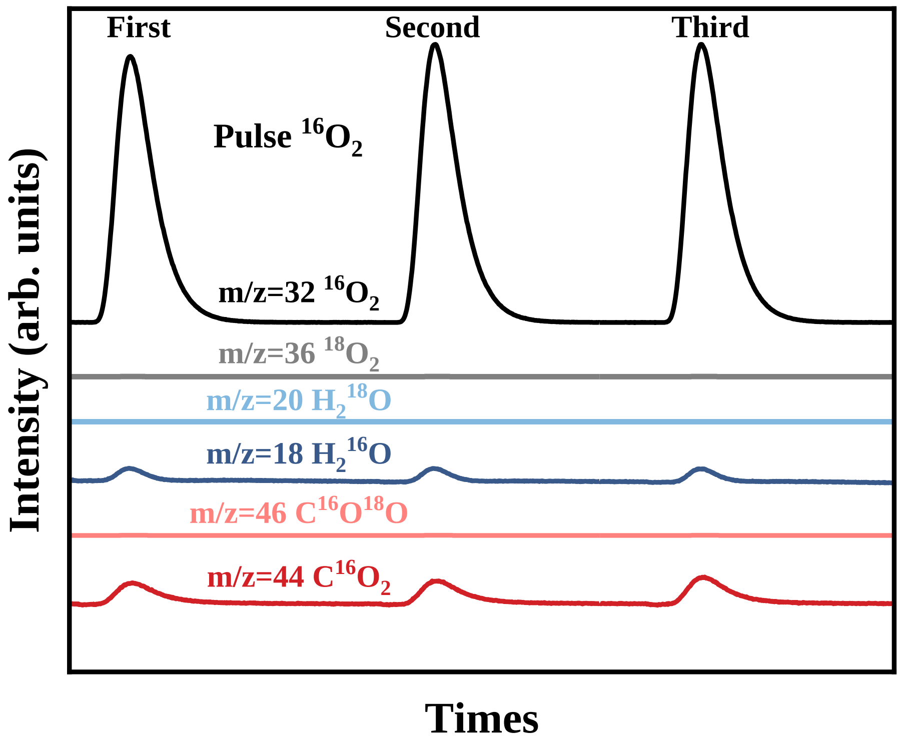

Supplementary Fig. 24. *In situ* O_2 pulse profiles of Pd/CeO_2-AT-Steam $H_2{}^{18}O$ catalyst, revealing that there is no oxygen-exchange on the catalyst during HCHO oxidation.

Supplementary Notes: Pd_1/CeO_2-AT sample was first treated in $H_2{}^{18}O$ at 750°C for 9 h to obtain the Pd/CeO_2-AT-Steam $H_2{}^{18}O$ catalyst. The obtained sample was placed in a U-tube of a homemade instrument for an O_2 pulse experiment. Briefly, a mixture of HCHO/He (10 mL·min^{-1}) was fed to the Pd/CeO_2-AT-Steam $H_2{}^{18}O$ catalyst at 30°C until the baseline reached flat, followed by the injection of the $^{16}O_2$ (99%; 0.3 mL each time) into the flow via a syringe. The chemical and isotopic compositions of the reactor effluent were measured by an online Pfeiffer Omni Star GSD 320 quadrupole mass spectrometer (QMS), equipped with a Secondary Electron Multiplier (SEM) detector. The m/z signals of 18, 20, 32, 36, 44, and 46 represent $H_2{}^{16}O$, $H_2{}^{18}O$, $^{16}O_2$, $^{18}O_2$, $C^{16}O_2$, and $C^{16}O^{18}O$, respectively.

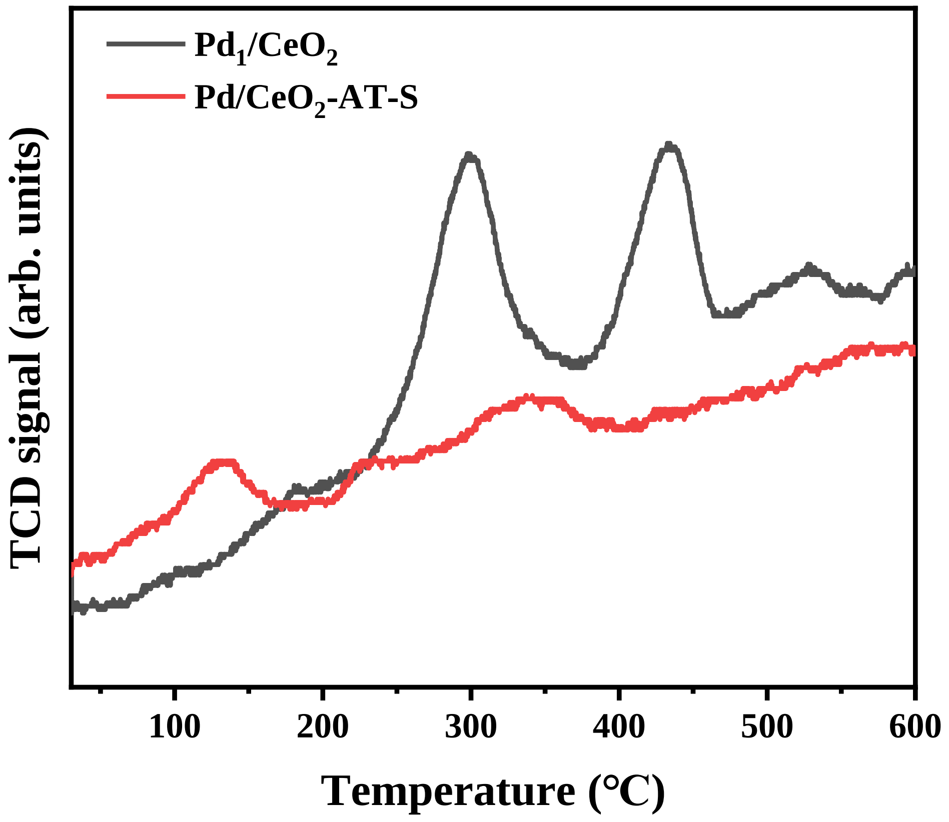

Supplementary Fig. 25. O_2 temperature-programmed reduction (O_2-TPD) profiles of the Pd/CeO_2-AT-S and Pd_1/CeO_2- AT catalysts.

Supplementary Note: As for Pd_1/CeO_2-AT, the desorption peaks of surface oxygen are concentrated at 300 °C and 450 °C. Likewise, for the Pd/CeO_2-AT-S catalyst, the surface reactive oxygen species begin to be gradually desorbed at 30°C. It indicated that the Pd/CeO_2-AT-S catalyst was more likely to adsorb activated O_2, which should play a key role to fulfill the catalytic oxidation of HCHO at low temperatures.

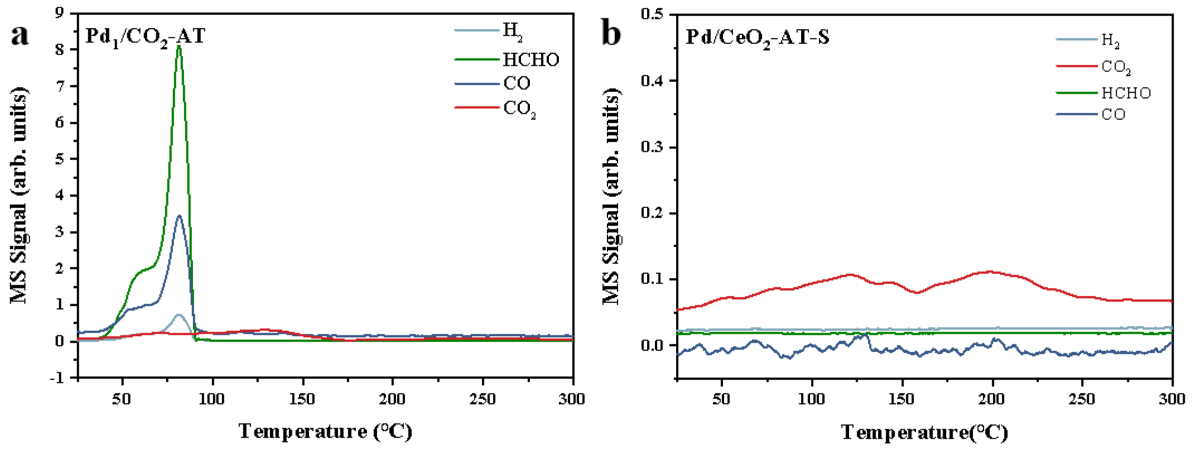

Supplementary Fig. 26. HCHO-TPSR profiles over Pd_1/CeO_2-AT (a) and Pd/CeO_2-AT-S (b) catalysts.

Supplementary Note: HCHO temperature programmed surface reaction (HCHO-TPSR) experiments were performed to evaluate HCHO oxidation as a function of temperature (30-300℃) on Pd_1/CeO_2-AT and Pd/CeO_2-AT-S, respectively. On the Pd_1/CeO_2-AT catalyst (**Supplementary Fig. 26a**), as the temperature increases to 42°C, most of the adsorbed formaldehyde begins to desorb, and a certain amount of CO and H_2 is also detected, probably due to the direct dehydrogenation pathway (HCHO → H_2 + CO), and it is not until 80℃ that a few CO_2 production from oxidation of surface HCHO starts to occur. Both CO and H_2 were also detected, probably due to the direct dehydrogenation route (HCHO → H_2 + CO). By contrast, the production of CO_2 took place even at room temperature over Pd/CeO_2-AT-S catalyst, no obvious CO and H_2 were observed over the entire temperature range studied (**Supplementary Fig. 26b**). However, on the Pd/CeO_2-AT-S catalyst, the CO_2 signal is much lower than that on the Pd_1/CeO_2-AT catalyst. This is because most of the HCHO is oxidized at room temperature, before the introduced O_2/He reaches a stable baseline. The drastic enhancement of reactivity brought by steam treatment is in good agreement with the earlier activity tests shown in **Fig. 1a**.

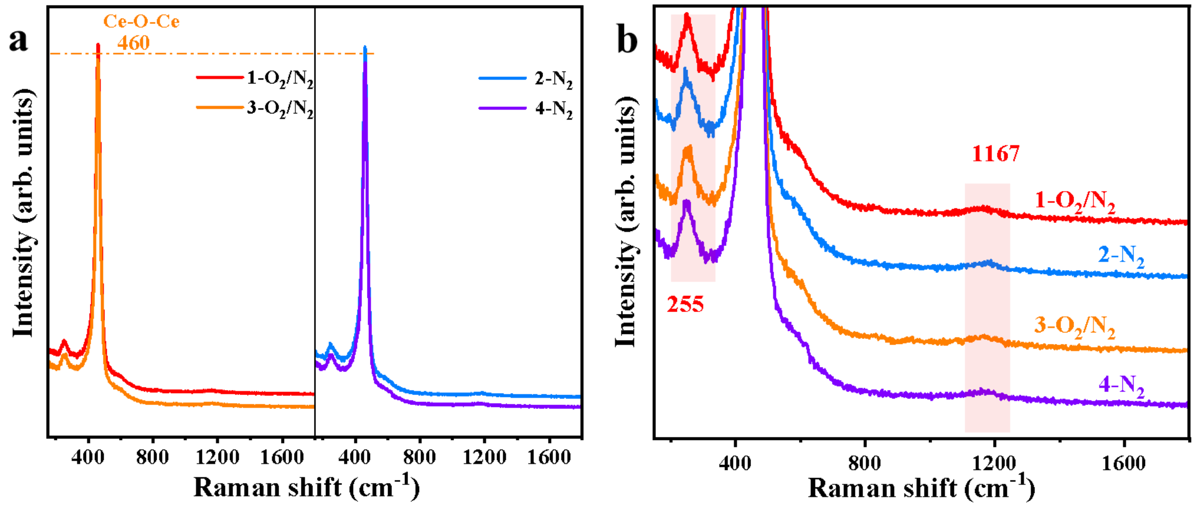

Supplementary Fig. 27. *In situ* Raman spectra (**a**) and the magnified spectra (**b**) of the Pd/CeO_2 nanoparticle catalyst cycled in different atmospheres (O_2/N_2 or N_2) at 120°C.

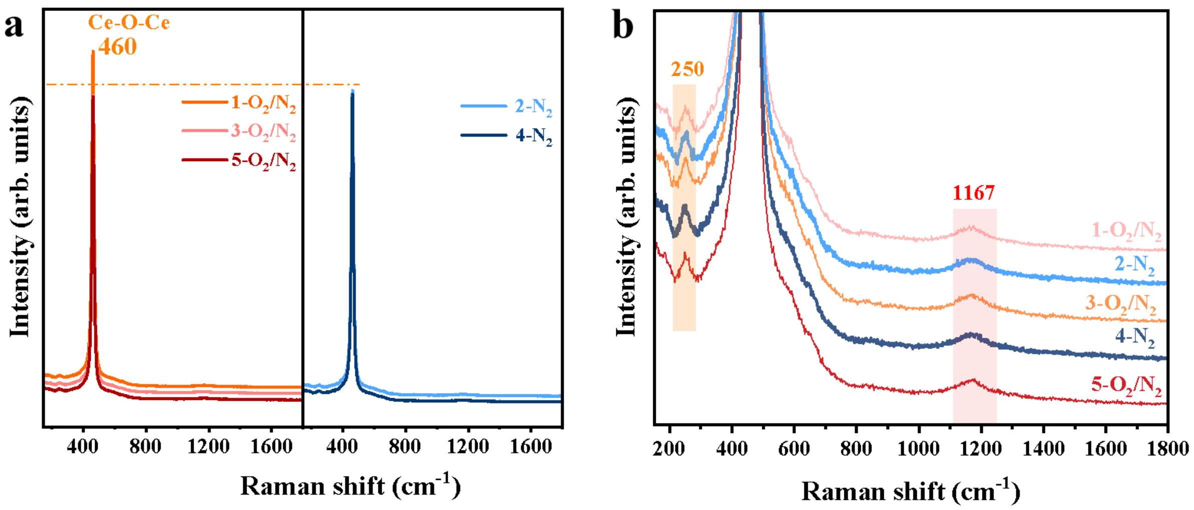

Supplementary Fig. 28. *In situ* Raman spectra (**a**) and magnified spectra (**b**) of the Pd_1/CeO_2-AT catalyst cycled in different atmospheres (O_2/N_2 or N_2) at 120°C.

Supplementary Table 5. The assignments of species over Pd/CeO_2 catalysts were observed in HCHO adsorption and reaction.

Species	Vibrational modes	IR bands wavenumber/cm^{-1} [7, 8, 9]	
		**Pd/CeO_2-AT-S**	**Pd_1/CeO_2-AT**
DOM[a]	ν(OCO)	1043, 1073, 1120	1102, 1125, 1170, 1006
	ν(CO)	933, 955	947,
HCOO[b]	ν_{as}(OCO)	1560, 1591	1585
	δ(CH)	1376	1372
	ν_s(OCO)	1322, 1360	1342, 1404
C-H (HCOO/DOM)	ν_s(OCO)+δ(CH) ν(CH)	2713, 2744, 2825, 2845, 2881	2748, 2813, 2840, 2852, 2897
OH (Ⅰ)[c]	ν(OH)		3694
OH (Ⅱ)[d]	ν(OH)	3667	
OH (Ⅲ)[e]	ν(OH)	3587	

[a] DOM = dioxymethylene

[b] HCOO = formate species

[c] OH (Ⅰ) = one-coordinated OH species

[d] OH (Ⅱ) = two-coordinated OH species

[e] OH (Ⅲ) = three-coordinated OH species

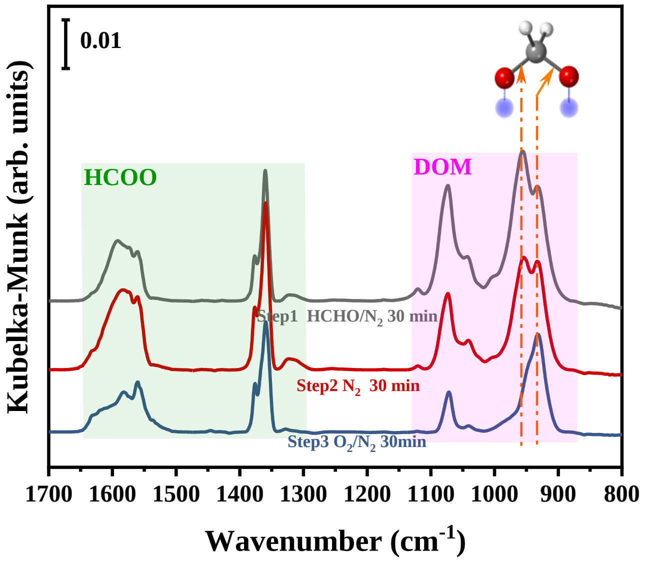

Supplementary Fig. 29. *In situ* HCHO-DRIFTS of the Pd/CeO_2-AT-S catalyst after exposure to a flow of HCHO/N_2 for 30 min (step 1), followed by N_2 degassing for 30 min (step 2), and followed by O_2/N_2 degassing for 30 min (step 3) at 30℃ (Insert picture: the structures of dioxymethylene).

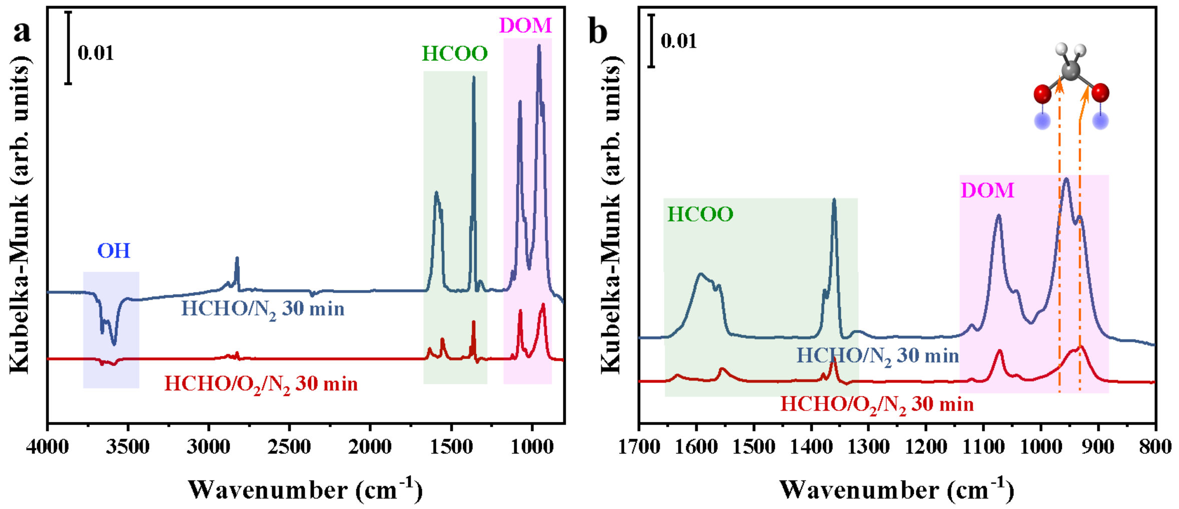

Supplementary Fig. 30. *In situ* HCHO-DRIFTS (**a**) and magnified spectra (**b**) of the Pd/CeO_2-AT-S catalyst exposure to a flow of HCHO/N_2 or HCHO/O_2/N_2 for 30 min at 30℃. (Inset picture: the structures of dioxymethylene)

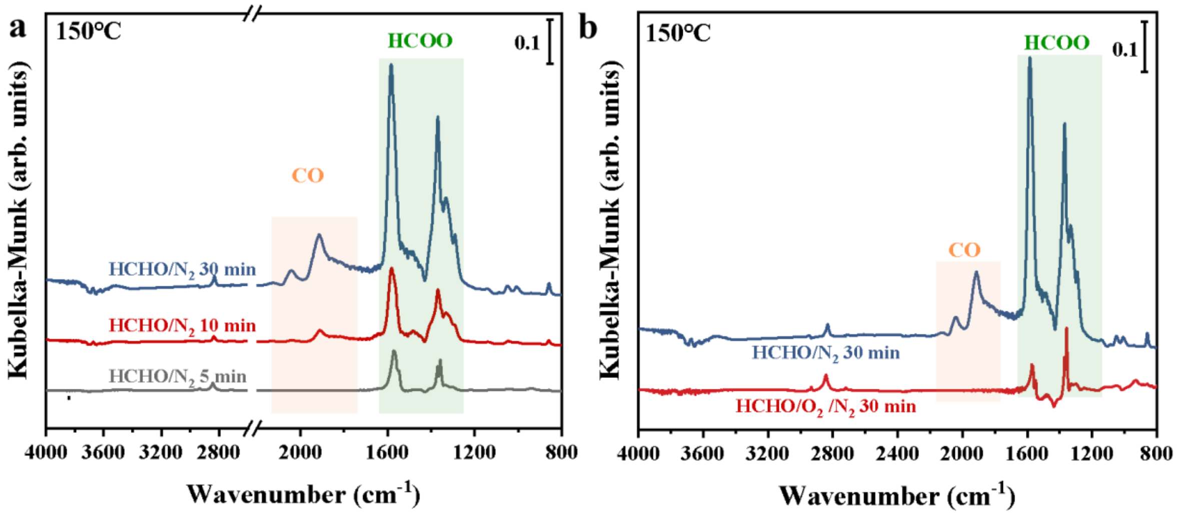

Supplementary Fig. 31. *In situ* HCHO-DRIFTS of the Pd_1/CeO_2-AT catalyst as a function of time in a flow of $HCHO/N_2$ at 150°C (**a**) and exposure to a flow of $HCHO/N_2$ or $HCHO/O_2/N_2$ for 30 min at 150°C (**b**).

Supplementary Table 6 Different structural models for Pd_1/step and Pd_1/terrace.

	**Pd_1/CeO_2-AT**	**Pd_1/CeO_2-I**
**Structural model**	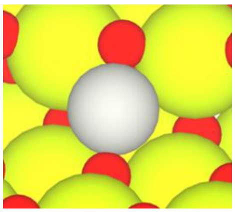<br>**Pd_1/step**	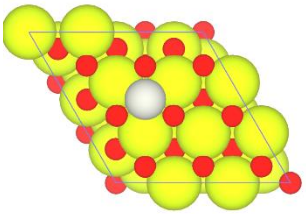<br>**Pd_1/terrace**
**Pd-O bond length/Å**	**2.02, 2.02**	**2.40, 2.40, 2.40, 2.88**
**Pd binding energy/eV**	**-3.77**	**-2.15**
**Migration model**	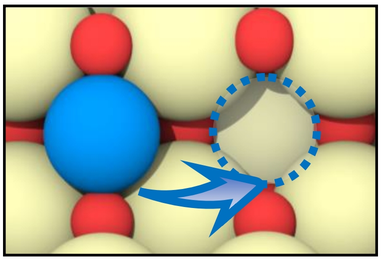	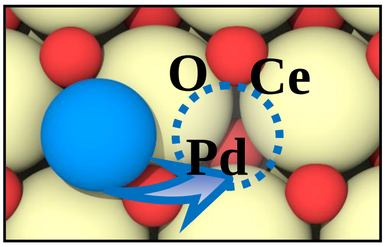
**Migration barrier /eV**	**1.45**	**0.14**

Supplementary Note: As reported in the previous work, the Pt located on the step site is more stable in the Pt_1/CeO_2 system[10, 11]. We therefore used this structure to perform the Pd_1/CeO_2 model and compared the binding energy and migration energy of Pd atom in the single-atom model of the step position and terrace position. We found that Pd is more stable in the step position than in the terrace (Supplementary Table 6). Combined with the experimental results, the Pd single-atom located at the CeO_2 step is regarded as the model of the Pd_1/CeO_2-AT catalyst prepared via the atom trapping method[12].

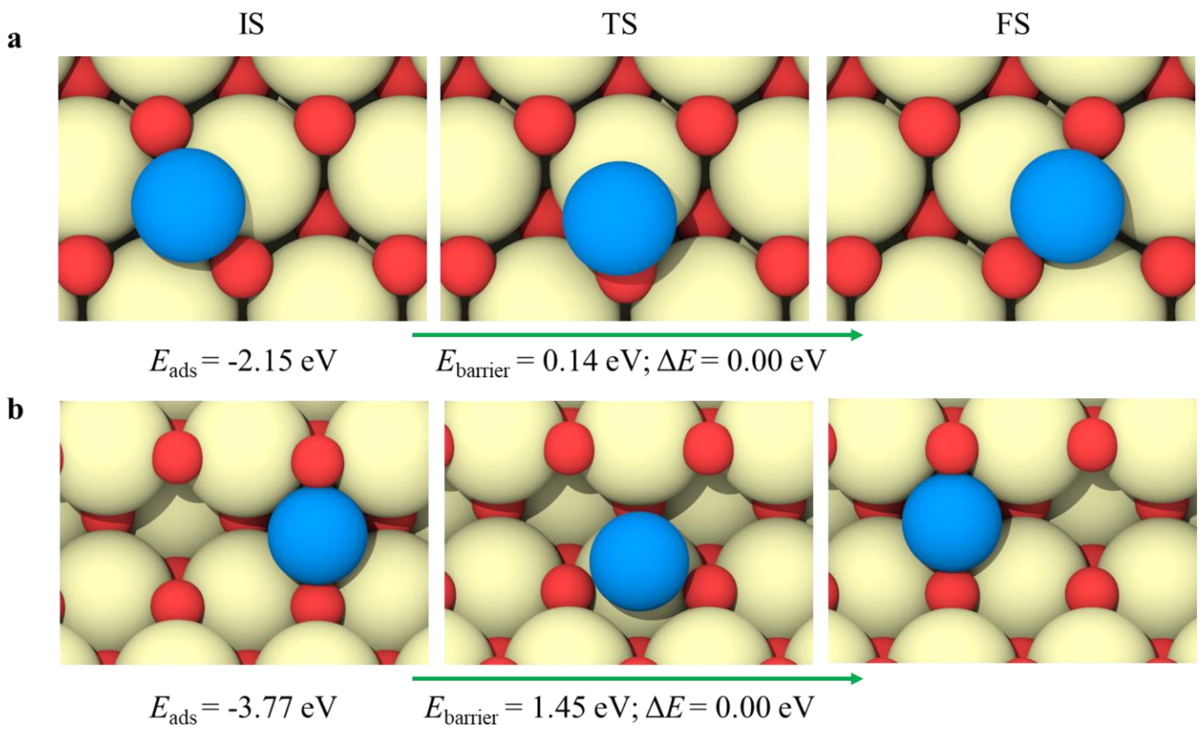

Supplementary Fig. 32. The initial state (IS), transition state (TS) and final state (FS) of Pd_1 migration over the (**a**) terrace and the (**b**) step of CeO_2 (111). Yellow, red and blue circles denote Ce, O and Pd atoms, respectively.

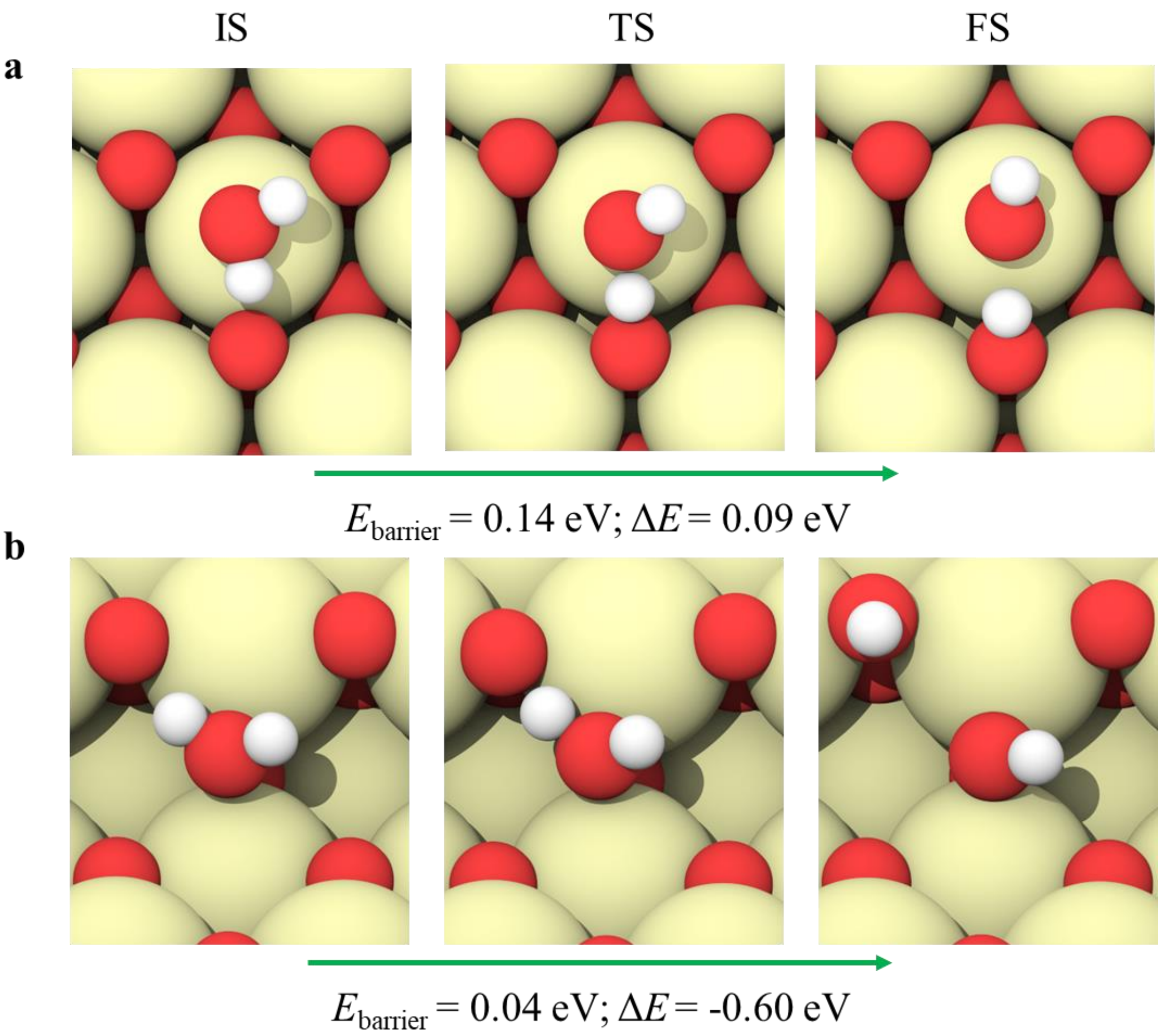

Supplementary Fig. 33. The initial state (IS), transition state (TS) and final state (FS) of H_2O dissociation over the CeO_2 (111) (**a**) terrace and (**b**) step. Yellow, red and white circles denote Ce, O and H atoms, respectively.

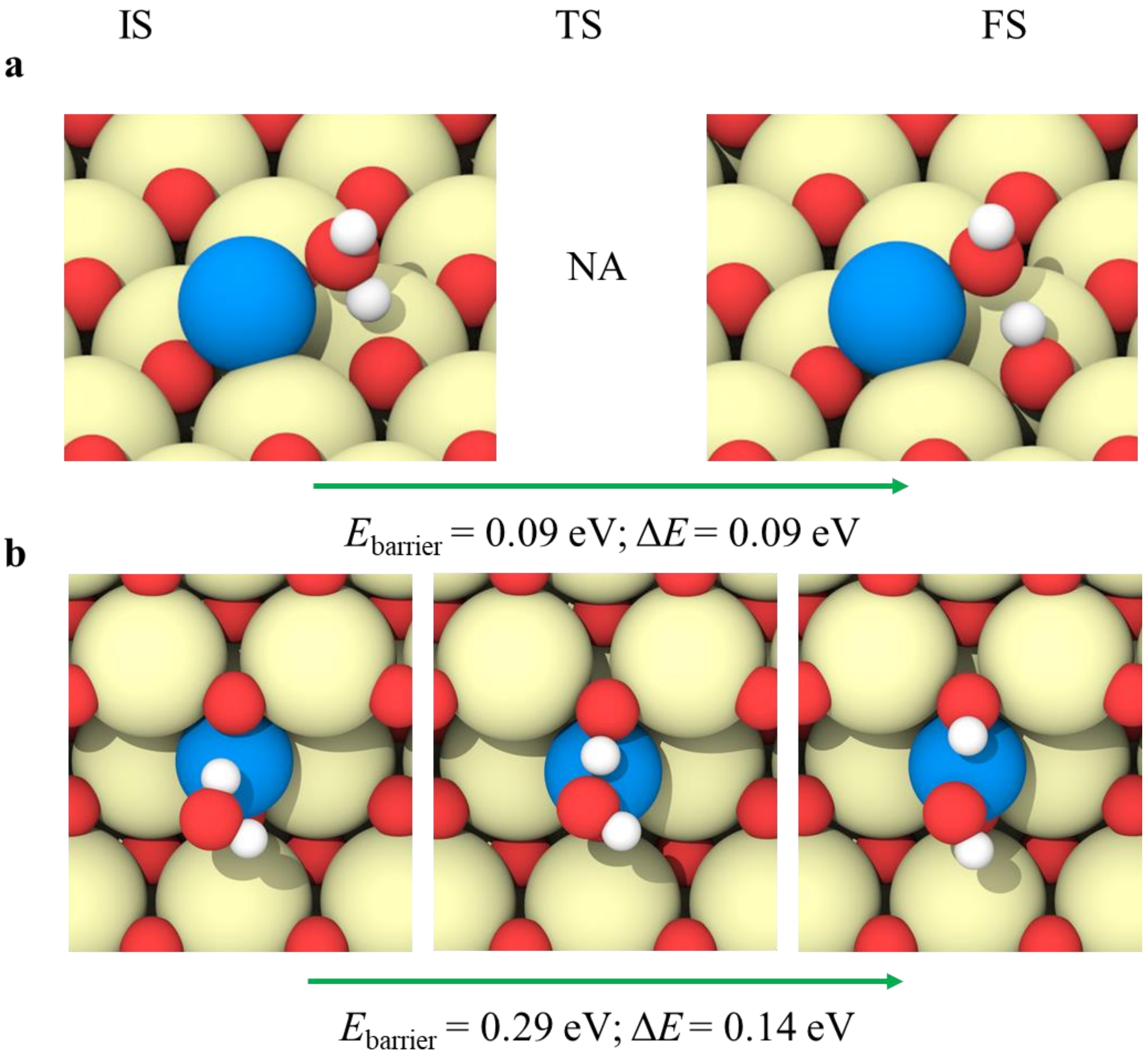

Supplementary Fig. 34. The initial state (IS), transition state (TS) and final state (FS) of H_2O dissociation over the (**a**) Pd_1/terrace and (**b**) Pd_1/step. Yellow, red, white, and blue circles denote Ce, O, H and Pd atoms, respectively.

Supplementary Notes: For the H_2O dissociation over Pd_1/terrace, the CI-NEB calculation result shows that the dissociation process has no TS and the cleavage of O-H of H_2O is an energy-increasing process.

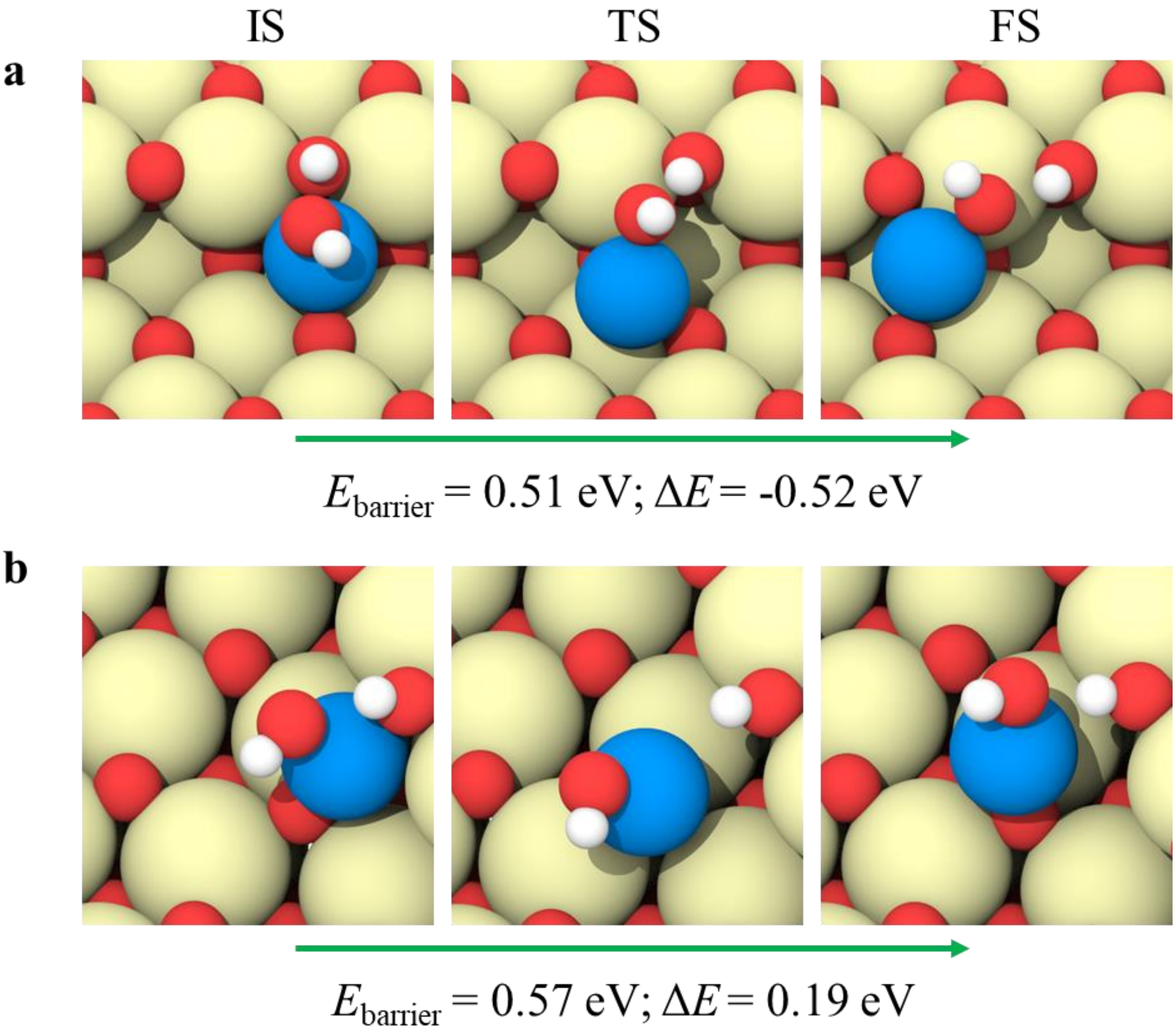

Supplementary Fig. 35. The initial state (IS), transition state (TS) and final state (FS) of Pd_1 migration (**a**) along the step direction and (**b**) towards the terrace site on the CeO_2 (111) step in the presence of H_2O. Yellow, red, white, and blue circles denote Ce, O, H and Pd atoms, respectively.

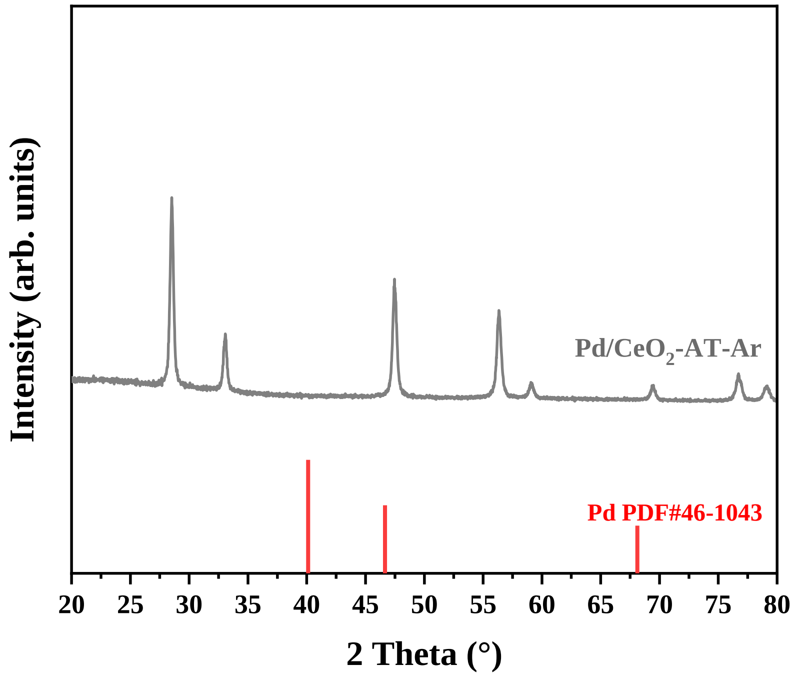

Supplementary Fig. 36. XRD pattern of the Pd/CeO_2-AT-Ar catalyst performed at a scanning speed of 2θ = 10 °/min from 20° to 80°. The Pd/CeO_2-AT-Ar catalyst was obtained by treating Pd_1/CeO_2-AT in argon at 750°C. The absence of metal Pd in the XRD pattern of the Pd/CeO_2-AT-Ar catalyst indicated that no very large Pd particle was presented.

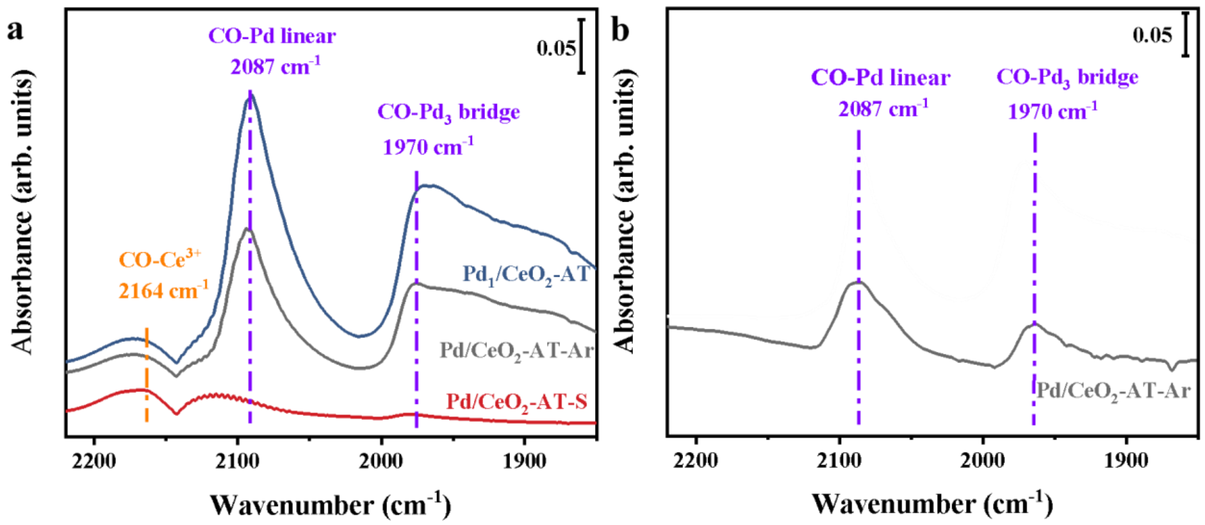

Supplementary Fig. 37. (**a**) CO-DRIFTS spectra of the Pd_1/CeO_2-AT, Pd/CeO_2-AT-Ar, and Pd/CeO_2-AT-S catalysts after exposure to a flow of $CO/O_2/N_2$ for 30 min at 30℃. (**b**) CO-DRIFTS spectra of the Pd/CeO_2-AT-Ar catalyst after exposure to a flow of $CO/O_2/N_2$ for 30 min and followed by O_2/N_2 degassing for 15 min at 30℃.

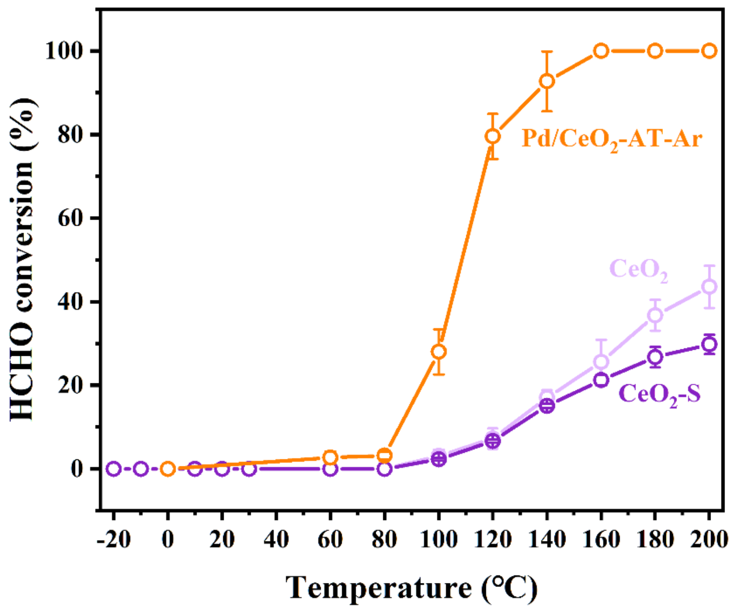

Supplementary Fig. 38. HCHO conversion as a function of temperature on supports and catalysts (CeO_2, CeO_2-S, and Pd/CeO_2-AT-Ar). The error bar indicates the standard deviation of the measured data (three different runs). Reaction condition: 400 ppm HCHO, 20 vol% O_2, and N_2 as balance gas, total flow rate: 50 mL·min^{-1}, WHSV (weight hourly space velocity): 150,000 mL·g^{-1}·h^{-1}. The Pd_1/CeO_2-AT catalyst treated in Ar (Pd/CeO_2-AT-Ar) shows significantly lower activity than that treated in steam.

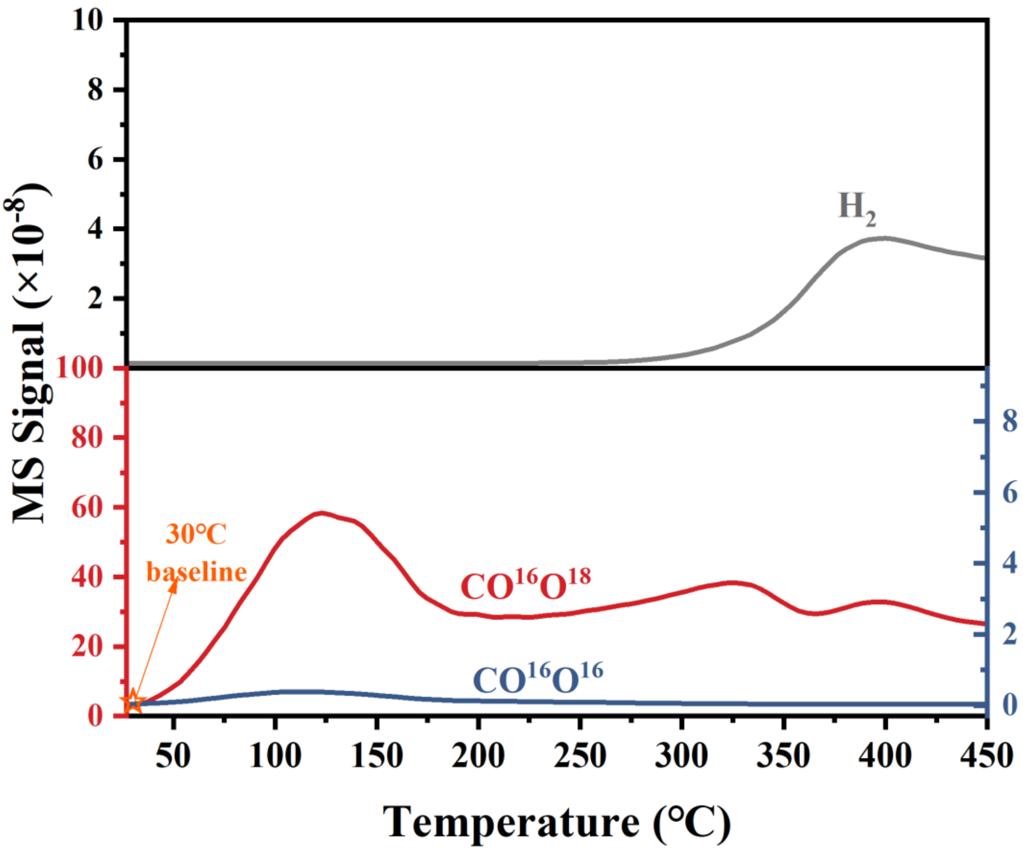

Supplementary Fig. 39. CO temperature-programmed reduction (CO-TPR) profiles of the Pd/CeO_2-AT-S catalyst after treating Pd_1/CeO_2-AT in H_2O^{18} at 750°C.

Supplementary Table 7. Detachment energy (eV) of Ce-O unit on the various surfaces (Values in parentheses are relative to the detachment of the same unit from the bare terrace).

**CeO**	**terrace**	**step**	**Pd_1/terrace**	**Pd_1/step**
In the absence of H_2O	13.06 (0.00)	10.89 (-2.17)	8.38 (-4.68)	9.29 (-3.77)
In the presence of H_2O	11.62 (-1.44)	9.48 (-3.58)	10.17 (-2.89)	8.01 (-5.05)

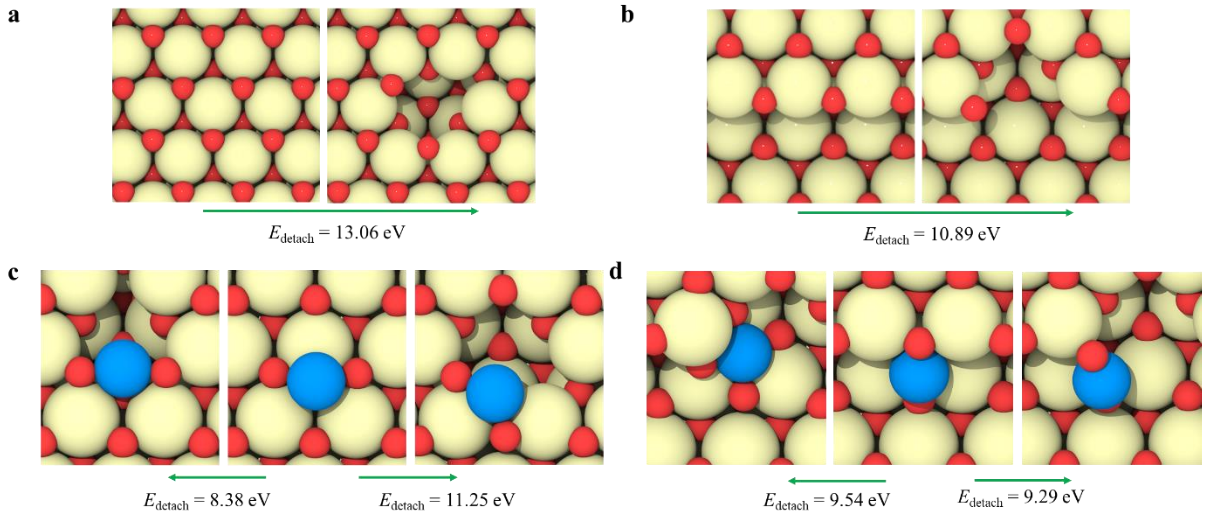

Supplementary Fig. 40. Detachment of Ce-O unit from the CeO_2 (111) (**a**) terrace, (**b**) step, (**c**) Pd_1/terrace and (**d**) Pd_1/step in the absence of H_2O. Yellow, red and blue circles denote Ce, O and Pd atoms, respectively. For one process with different products (e.g., **c** and **d**), the corresponding lowest value of detachment energy was listed in **Supplementary Table 7.**

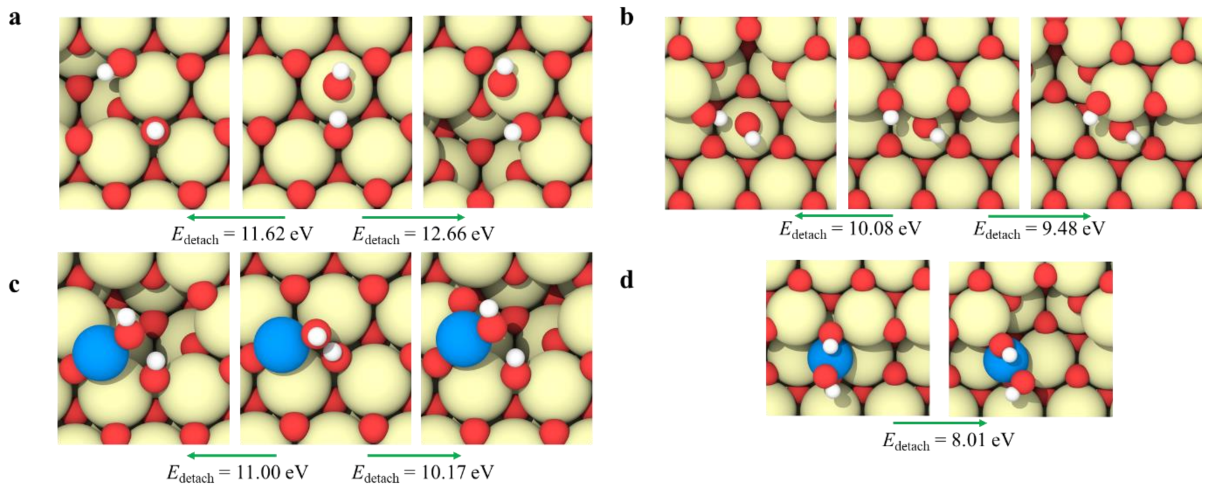

Supplementary Fig. 41. Detachment of Ce-O unit from CeO_2 (111) (**a**) terrace, (**b**) step, (**c**) Pd_1/terrace, and (**d**) Pd_1/step in the presence of H_2O. Yellow, red, white, and blue circles denote Ce, O, H and Pd atoms, respectively.

Supplementary Table 8. Detachment energy (eV) of Pd_1(in the absence of H_2O) or Pd_1-OH (surfaces in the presence of H_2O) unit on the various surfaces (Values in parentheses are relative to the detachment of same unit from the Pd_1/terrace).

**Pd_1/Pd_1-OH**	**Pd_1/terrace**	**Pd_1/step**
In the absence of H_2O	2.15 (0.00)	3.77 (1.62)
In the presence of H_2O	-0.30 (-2.45)	2.71 (0.56)

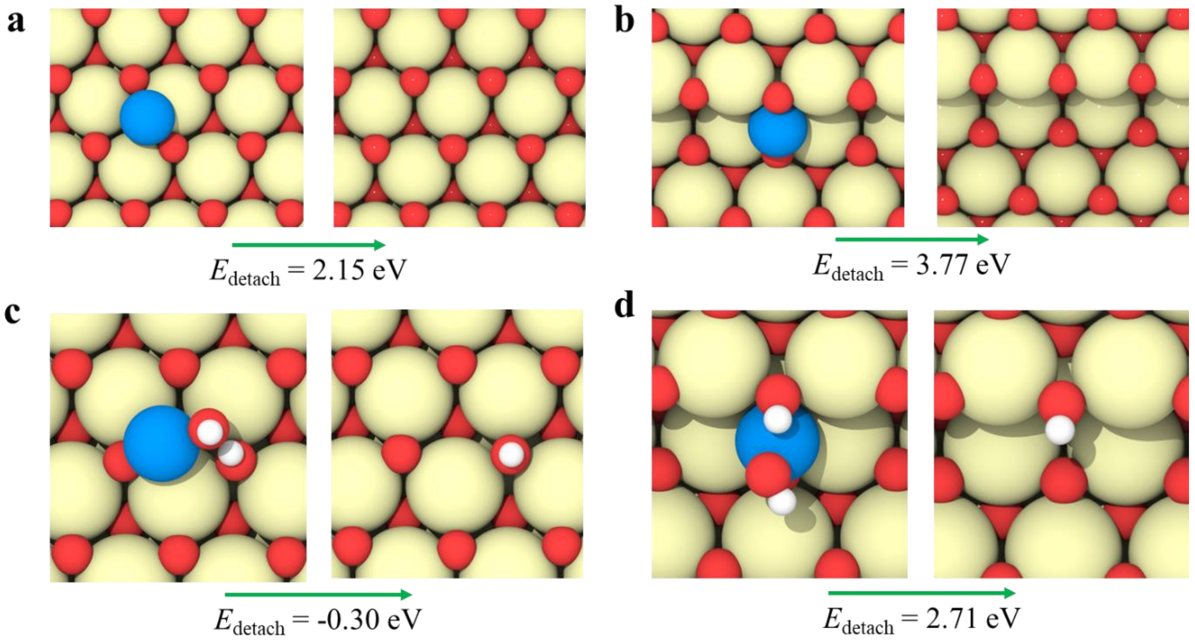

Supplementary Fig. 42. Detachment of Pd_1 unit from the (**a**) Pd_1/terrace and (**b**) Pd_1/step in the absence of H_2O. Detachment of Pd_1-OH from the (**c**) Pd_1/terrace and (**d**) Pd_1/step in the presence of H_2O. Yellow, red, white, and blue circles denote Ce, O, H and Pd atoms, respectively.

Supplementary Table 9. Detachment energy (eV) of Pd_1CeO (in the absence of H_2O) or $HOPd_1$-CeOH (in the presence of H_2O) unit on the various surfaces (Values in parentheses are relative to the detachment of the same unit from the Pd_1/terrace).

**Pd_1CeO/$HOPd_1$-CeOH**	**Pd_1/terrace**	**Pd_1/step**
In the absence of H_2O	12.80 (0.00)	12.24 (-0.55)
In the presence of H_2O	13.23 (0.43)	11.78 (-1.02)

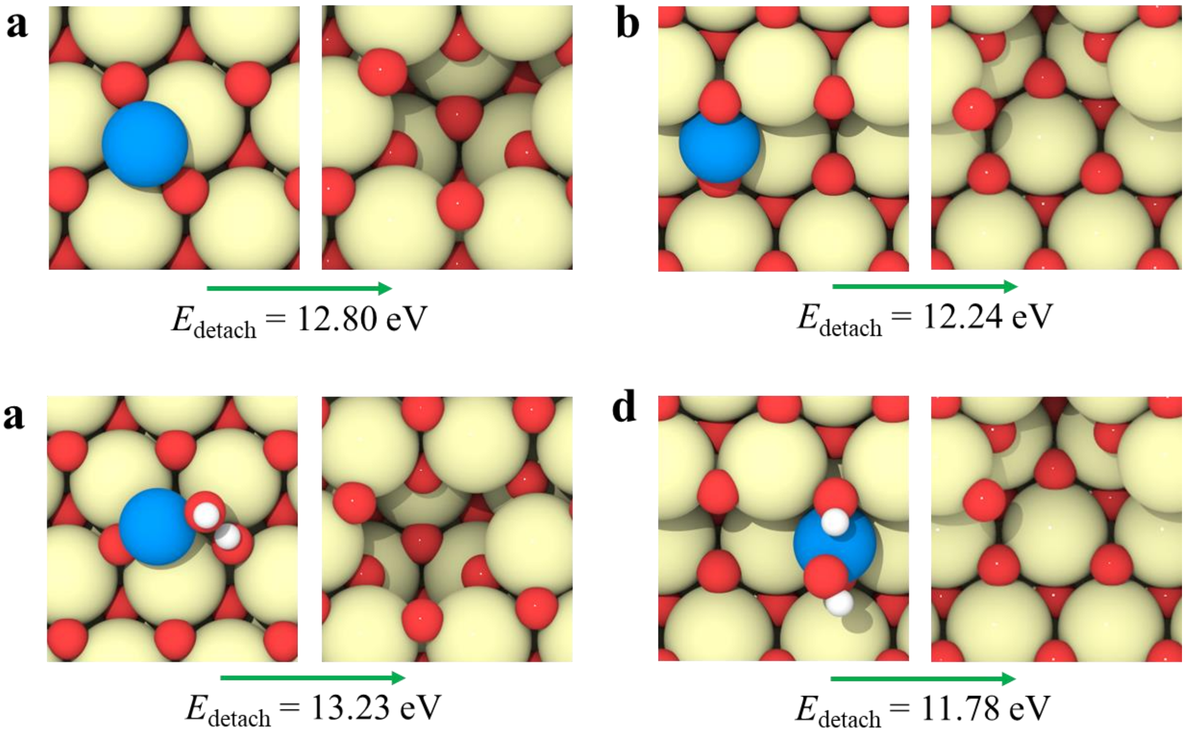

Supplementary Fig. 43. Detachment of Pd_1-CeO unit from the (**a**) Pd_1/terrace and (**b**) Pd_1/step in the absence of H_2O. Detachment of $HOPd_1$-CeOH from the (**c**) Pd_1/terrace and (**d**) Pd_1/step in the presence of H_2O. Yellow, red, white, and blue circles denote Ce, O, H and Pd atoms, respectively.

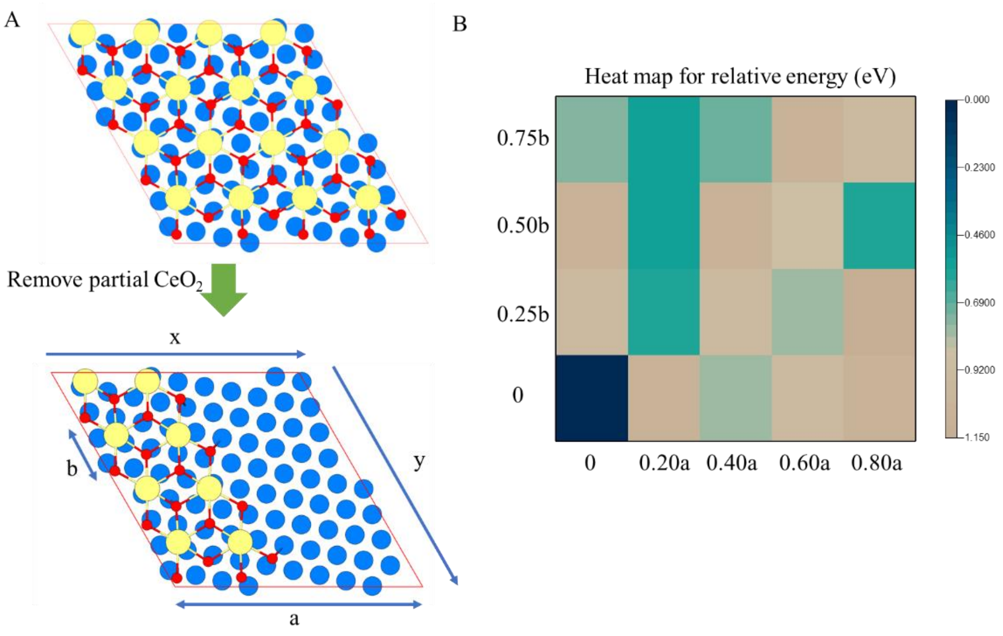

Supplementary Fig. 44. (**A**) Scheme of the construction of Pd-CeO_2 domain interface. (**B**) Heat map for relative energy of various interface structures.

Supplementary Notes: The optimum location of the residual CeO_2 was determined by comparing the total energy of various optimized structures deviating from the continuously moving the residual CeO_2 ribbon along x and y. The total displacement in the y direction is equal to the distance between the two O (b in **Supplementary Fig. 44A** bottom), while in the x direction is the length of the adopted supercell vector (a).

Supplementary Table 10. Bader charge (Q_{Ce}/|e|) of Ce in the constructed Ce_2O_3-Pd interface, CeO_2 bulk and Ce_2O_3 bulk.

	**interface**	**CeO_2**	**Ce_2O_3**
Q_{Ce}	+2.18	+2.44	+2.04

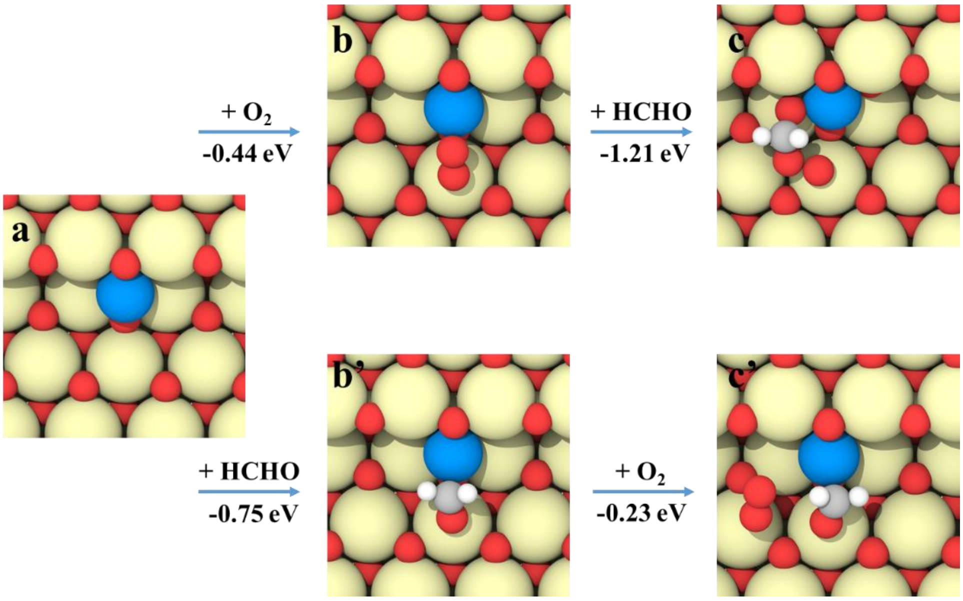

Supplementary Fig. 45. Adsorption of HCHO/O_2 reactants on Pd_1/step.

Supplementary Notes: Adsorption of reactants (HCHO and O_2) on Pd_1/step was investigated. One can see from **Supplementary Fig. 45** that the O_2 and HCHO adsorption on Pd_1/step is weaker than that at the interface (-1.57 and -1.87 eV), as indicated in **Fig. 4c** of the manuscript. More importantly, the dehydrogenation of HCHO in structure c first undergoes cleavage of the O-O bond, encountering an energy barrier of 1.51 eV. These results suggested that the oxidation of HCHO is not favored via the co-adsorption of species, which can be attributed to the weaker adsorption and activation of O_2 on Pd_1/step. Yellow, red, white, gray and blue circles denote Ce, O, H, C and Pd atoms, respectively.

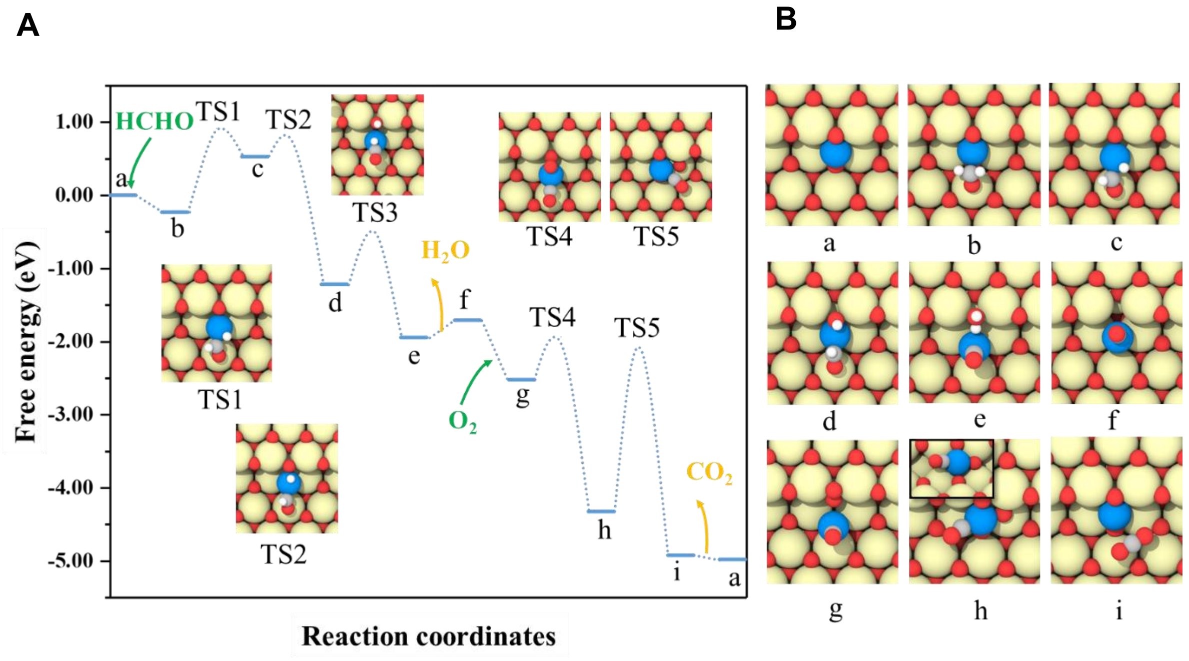

Supplementary Fig. 46. (A) A proposed reaction pathway of HCHO oxidation on Pd_1/step. Yellow, red, white, gray and blue circles denote Ce, O, H, C and Pd atoms, respectively. (B) The configuration during the dehydrogenation of HCHO by O from Pd_1/step.

Supplementary Notes: The dehydrogenation of HCHO by O from Pd_1/step was also investigated. Starting from the adsorption of HCHO at Pd_1 (a to b), *HCHO loses two H to form one CO molecule with an H_2O, the highest free energy barrier encountered in the process (b-e) is 1.15 eV. H_2O is then desorbed from the step, leaving an oxygen vacancy (e to f), followed by the adsorption of an O_2 molecule (g) and CO oxidation (g to i). The NEB calculation of the direct oxidation of CO by $*O_2$ encounters a minimum energy point (h), where $*O_2$ dissociates on the step and one of O's fills the O vacancy, while the other O goes to the step and binds to a Pd with two O's (h). Therefore, the O-O should be broken first owing to the high stability of state h. Moreover, the high stability of state h results in a free energy barrier up to 2.25 eV for CO oxidation, suggesting that the HCHO oxidation by lattice O on Pd_1/step is also difficult and shows lower performance than that of the interface.

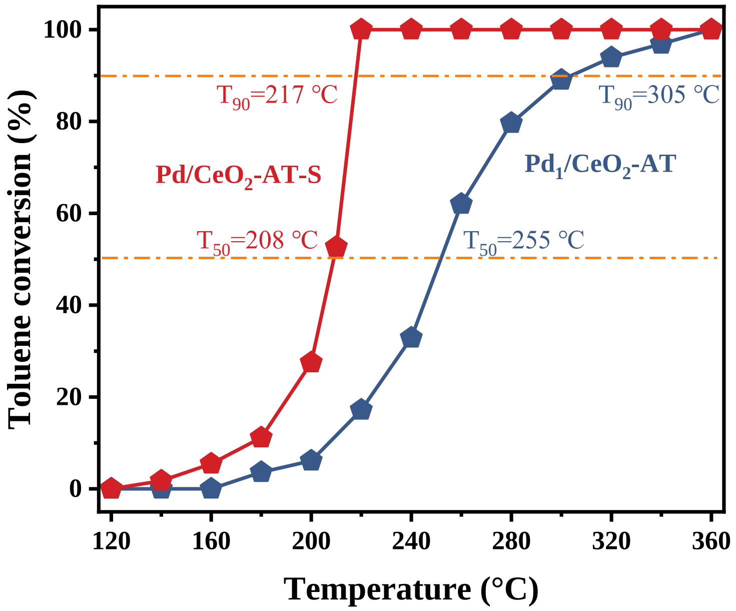

Supplementary Fig. 47 Toluene conversion as a function of temperature on catalysts (Pd_1/CeO_2-AT and Pd/CeO_2-AT-S) in toluene oxidation. Reaction condition: 1000 ppm toluene, 20 vol% O_2, and N_2 as balance gas, total flow rate: 60 mL·min^{-1}, WHSV (weight hourly space velocity): 18,000 mL·g^{-1}·h^{-1}.

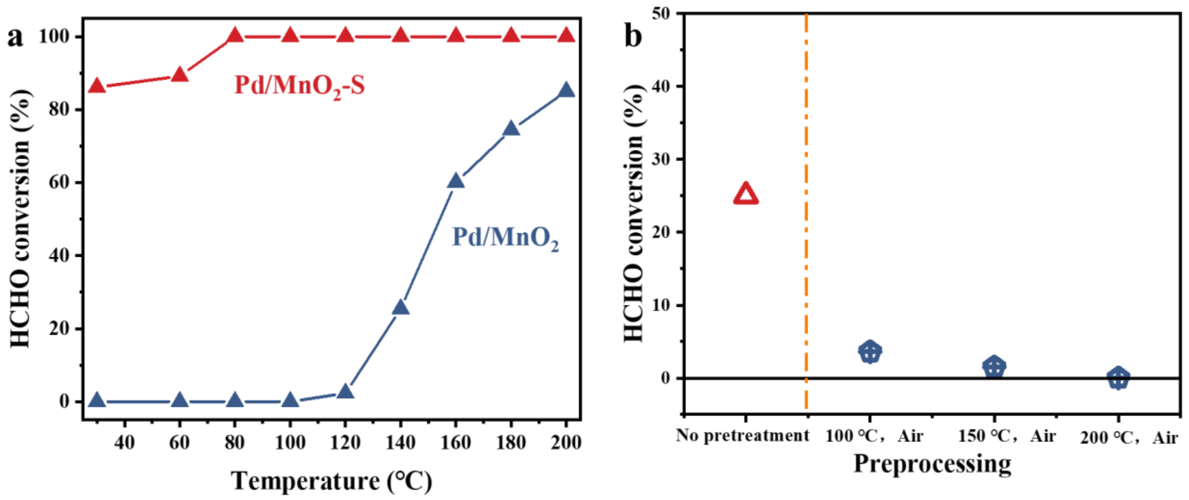

Supplementary Fig. 48. (**a**) HCHO conversion as a function of temperature on catalysts (Pd_1/MnO_2 and Pd/MnO_2-S). Reaction condition: 400 ppm HCHO, 20 vol% O_2, and N_2 as balance gas, total flow rate: 50 $mL{\cdot}min^{-1}$, WHSV (weight hourly space velocity): 12,000 $mL{\cdot}g^{-1}{\cdot}h^{-1}$. The Pd/MnO_2-S was achieved by steam treatment of Pd_1/MnO_2 at 750°C for 9 h. (**b**) HCHO conversion on the Pd/MnO_2-S catalyst treated at different oxidation temperatures. Reaction conditions: 30°C, 400 ppm HCHO, 20 vol% O_2, and N_2 as balance gas, and total flow rate: 50 $mL{\cdot}min^{-1}$, WHSV (weight hourly space velocity): 30,000 $mL{\cdot}g^{-1}{\cdot}h^{-1}$.

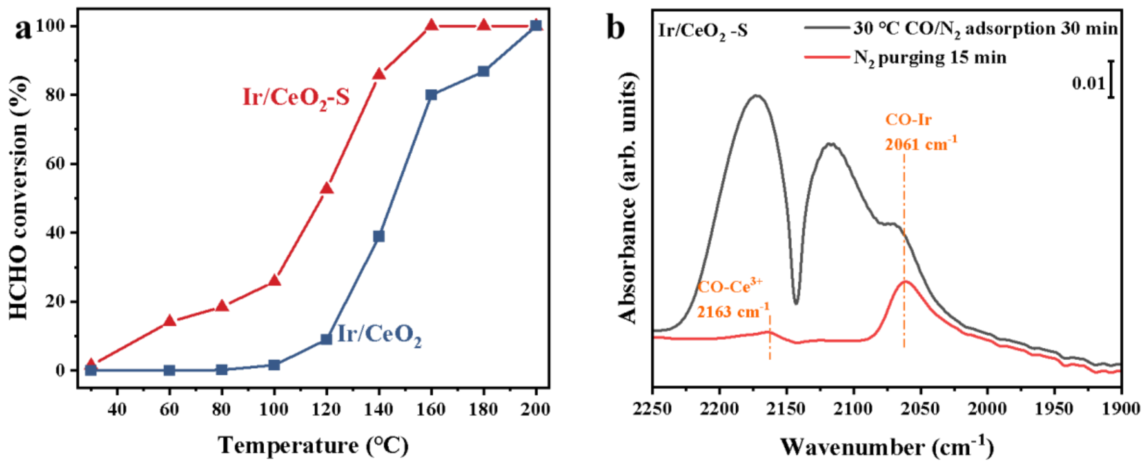

Supplementary Fig. 49. (**a**) HCHO conversion as a function of temperature on catalysts (Ir_1/CeO_2 and Ir/CeO_2-S). Reaction condition: 400 ppm HCHO, 20 vol% O_2, and N_2 as balance gas, total flow rate: 50 $mL\cdot min^{-1}$, WHSV (weight hourly space velocity): 60,000 $mL\cdot g^{-1}\cdot h^{-1}$. (**b**) CO-DRIFTS spectra of the Ir/CeO_2-S catalyst after exposure to a flow of $CO/O_2/N_2$ for 30 min, followed by degassing in O_2/N_2 for 15 min at 30°C. It shows the presence of CO peaks adsorbed on both Ce^{3+} and Ir nanoparticles.

Supplementary References

1. Wang C, *et al.* A simple strategy to improve Pd dispersion and enhance Pd/TiO_2 catalytic activity for formaldehyde oxidation: The roles of surface defects. *Applied Catalysis B: Environmental* **282**, 119540 (2021).

2. Xiang N, *et al.* Size effect of γ-Al_2O_3 supports on the catalytic performance of Pd/γ-Al_2O_3 catalysts for HCHO oxidation. *Molecular Catalysis* **494**, 111112 (2020).

3. Li Y, Zhang C, He H. Significant enhancement in activity of Pd/TiO_2 catalyst for formaldehyde oxidation by Na addition. *Catalysis Today* **281**, 412-417 (2017).

4. Tan H, Wang J, Yu S, Zhou K. Support morphology-dependent catalytic activity of Pd/CeO_2 for formaldehyde oxidation. *Environmental Science & Technology* **49**, 8675-8682 (2015).

5. Li GN, Li L. Highly efficient formaldehyde elimination over meso-structured M/CeO_2 (M = Pd, Pt, Au and Ag) catalyst under ambient conditions. *Rsc Advances* **5**, 36428-36433 (2015).

6. Jiang D, *et al.* Elucidation of the active sites in single-atom Pd_1/CeO_2 catalysts for low-temperature CO oxidation. *ACS Catalysis* **10**, 11356-11364 (2020).

7. Li C, Domen K, Maruya K, Onishi T. Spectroscopic identification of adsorbed species derived from adsorption and decomposition of formic acid, methanol, and formaldehyde on cerium oxide. *Journal of Catalysis* **125**, 445-455 (1990).

8. Ma L, Seo CY, Chen X, Li J, Schwank JW. Sodium-promoted Ag/CeO_2 nanospheres for catalytic oxidation of formaldehyde. *Chemical Engineering Journal* **350**, 419-428 (2018).

9. Ahmed Badri CB, Jean-Claude Lavalley An FTIR study of surface ceria hydroxy groups during a redox process with H_2. *Journal of the Chemical Society, Faraday Transactions* **92**, 4669-4673 (1996).

10. Pereira-Hernandez XI, *et al.* Tuning Pt-CeO_2 interactions by high-temperature vapor-phase synthesis for improved reducibility of lattice oxygen. *Nature communications* **10**, 1358 (2019).

11. Jones J, *et al.* Thermally stable single-atom platinum-on-ceria catalysts via atom trapping. *Science* **353**, 150-154 (2016).

12. Kunwar D, *et al.* Stabilizing high metal loadings of thermally stable platinum single atoms on an industrial catalyst support. *ACS Catalysis* **9**, 3978-3990 (2019).
